# Adaptive Twisting Metamaterials

**DOI:** 10.1002/adma.202513714

**Published:** 2025-10-22

**Authors:** Mattia Utzeri, Maria L. Gatto, Edoardo Mancini, Donato Orlandi, Daniele Cortis, Marco Sasso, Shanmugam Kumar

**Affiliations:** ^1^ Department of Industrial Engineering and Mathematical Sciences Polytechnic University of Marche Ancona 60121 Italy; ^2^ Department of Industrial and Information Engineering and Economics University of L'Aquila L'Aquila 67100 Italy; ^3^ Gran Sasso National Laboratory National Institute for Nuclear Physics Assergi 67100 Italy; ^4^ James Watt School of Engineering University of Glasgow Glasgow G128QQ UK

**Keywords:** adaptive crashworthiness, additive manufacturing, Cosserat continuum mechanics, twisting gyroid, twisting metamaterials

## Abstract

Next‐generation protective systems require adaptive materials capable of reconfiguring their response to impact type and severity, thereby offering multiple force–displacement pathways. Here, the study introduces twisting metamaterials, a subclass of architected lattices whose mechanics are captured by micropolar elasticity. Derived from twisting operations on primitive lattices, these structures exhibit geometry‐induced torsional actuation and nonlinear responses, enabling adaptive crashworthiness. A multiscale predictive framework—combining Cosserat continuum mechanics, finite element modeling, and experiments—demonstrates its viability. Twisting sheet‐based gyroid structures (10% relative density) are additively manufactured in FE7131 steel and tested under quasi‐static and dynamic compression with varied torsional constraints, revealing adaptive energy absorption. When rotation is constrained, the structures achieve high axial stiffness (4.8 GPa), collapse stress (21 MPa), and specific energy absorption (15.36 J g^−1^), while free‐to‐twist and over‐rotation conditions reduce these values by up to 25%, 24%, and 33%, respectively. A macroscale model captures both axial and torsional responses, while SEM and µCT analyses of process‐induced defects inform a parametric finite element study extended to 5% and 15% relative densities. Mapping their performance onto an Ashby chart highlights twisting metamaterials as a promising class of mechanically adaptive, crashworthy materials for advanced protection systems in automotive, rail, aerospace, and defence applications.

## Graphical abstract

This work introduces torque‐controlled twisting metamaterials as a transformative platform for adaptive crashworthiness. By combining multiscale predictive modeling with experimental validation on additively manufactured gyroids, it demonstrates tunable stiffness, collapse stress, and energy absorption. These architected materials enable real‐time mechanical adaptability, representing a paradigm shift in smart impact protection for transportation and defense.

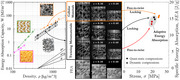

## 1 Introduction

The design of mechanical components for engineering applications has evolved significantly in recent years, driven by the demand for enhanced performance and versatility. Modern components must not only fulfill their primary mechanical functions but also meet a broad range of additional engineering requirements, including multifunctionality, ergonomics, sustainability, recyclability, comfort, safety, and compatibility with efficient manufacturing processes.^[^ ^1^, ^2^ ^]^ Looking ahead, next‐generation components are expected to transcend passive functionality by actively adapting to changing operational conditions and varying severity levels, while continuing to satisfy other engineering criteria.^[^ ^3^, ^4^ ^]^ This shift in design philosophy has led to the emergence of active components composed of adaptive materials, capable of dynamically adjusting their mechanical responses to external stimuli such as temperature,^[^ ^5^ ^]^ light,^[^ ^6^ ^]^ electromagnetic field,^[^ ^7^ ^]^ jamming,^[^ ^8^ ^]^ or interlocking networks.^[^ ^9^ ^]^

In the domain of impact absorption, progress in mechanical design is exemplified by innovations in the automotive sector.^[^ ^10^ ^]^ Early impact absorbers—such as crash boxes and bumpers—provided a single force–displacement response characteristic for all crash scenarios encountered during vehicle collisions. The early design philosophy emphasized material selection for the absorbing structure to strike a balance among competing performance requirements, seeking the optimum achievable compromise.^[^ ^11^ ^]^ However, the growing need to comply with increasingly stringent and often conflicting safety standards has shifted the focus toward the structural optimization of sacrificial components, advancing the philosophy of mechanical design. For example, in frontal collisions, the front structure of a vehicle must remain sufficiently rigid to prevent intrusion and protect the driver, while also being compliant enough to absorb crash energy and minimize injuries to pedestrians. Optimization efforts have included modifying structure geometry (e.g., shape,^[^ ^12^ ^]^ geometric gradients,^[^ ^13^ ^]^ and geometric sweeps^[^ ^14^ ^]^) or incorporating heterogeneous materials (e.g., multi‐material systems^[^ ^15^, ^16^, ^17^ ^]^) within proven energy absorbers to enhance energy dissipation. Despite their ingenuity, such approaches render the engineering solutions adaptable rather than adaptive, since the force–displacement response remains fixed—albeit optimized—for different impact scenarios.

By contrast, *adaptive* mechanical structures should respond to impacts of different types and severities by providing multiple force–displacement responses depending on external stimuli.^[^ ^3^, ^10^ ^]^ Through external actuation, adaptive structures can actively reconfigure their mechanical behavior to meet specific impact conditions with optimized performance. This trend toward active control aligns with emerging engineering paradigms in real‐time environmental monitoring^[^ ^18^ ^]^ and intelligent decision‐making systems that process contextual information and generate targeted mechanical responses.^[^ ^19^, ^20^ ^]^ As such, the development of adaptive materials for next‐generation protective systems is becoming increasingly essential, fuelling intense research across materials science, mechanics, and manufacturing disciplines.

Recent progress in adaptive materials has largely relied on experimental strategies, employing either bioinspired^[^ ^21^ ^]^ or rationally designed architectures^[^ ^22^ ^]^ that alter their mechanical response under external stimuli. However, most adaptive energy‐absorbing materials remain unsuitable for demanding engineering applications: soft systems such as hydrogels are mechanically too compliant,^[^ ^23^ ^]^ others depend on impractical actuation conditions such as intense electromagnetic or thermal fields,^[^ ^24^ ^]^ and many lack robust mesoscale predictive models.

Recently, Zhao et al.^[^ ^25^ ^]^ and Zang et al.^[^ ^26^ ^]^ modulated the force–displacement compression response of modular origami using external torque, demonstrating that torsional actuation provides a practical route for tuning mechanical behavior. Zhao et al. exploited compression–torsion coupling mechanical (CTCM) metamaterials, which intrinsically couple rotational and translational deformations due to their topological layout: two parallel planes connected by axisymmetric inclined struts, such that geometric chirality induces rotation.^[^ ^27^, ^28^, ^29^ ^]^ Comparable compression–torsion coupling can be realized using plate‐based Kresling origami,^[^ ^26^, ^30^, ^31^ ^]^ 3D chiral metamaterials with more sophisticated shapes,^[^ ^32^, ^33^, ^34^ ^]^ or short soft cylindrical shells operating in controllable buckling modes.^[^ ^35^ ^]^ While these studies introduced compression–torsion coupling through specific geometrical layouts (e.g., chirality, axisymmetric struts, origami folds, or cylindrical shells), here we propose twisting metamaterials as a new subclass of CTCM metamaterials. Twisting metamaterials can be derived by applying a twisting operation to primitive lattice geometries, providing a simple and generalizable design pathway to obtain CTCM metamaterials from generic lattices. As demonstrated by Frenzel et al,^[^ ^36^ ^]^ all CTCM metamaterials are governed by micropolar elasticity because their complex compression–torsion behavior can be captured using Cosserat continuum theory. This framework treats rotations and displacements independently, enabling the formulation of micropolar mesoscale models that are directly compatible with numerical simulations and supporting design and topological optimization of materials via micropolar constitutive parameters tailored to specific engineering needs.^[^ ^37^, ^38^, ^39^ ^]^ Therefore, Cosserat continuum mechanics offers a robust theoretical foundation to demonstrate the adaptive energy absorption performance of twisting metamaterials when actuation involves rotational motion, a practically implementable mechanical stimulus.

This study advances metamaterials research toward impact engineering by establishing twisting metamaterials as adaptive crashworthy materials through a predictive framework that integrates Cosserat continuum mechanics, finite element modeling, and experimental validation. Adaptive energy absorption is governed by torsional actuation, external torque, or rotational inertia, positioning twisting metamaterials as an engineering solution for next‐generation protection systems. The adaptivity concept is first formulated within the Cosserat continuum framework, focusing on axial stiffness, initial collapse stress, and specific energy absorption of a micropolar elastic material subjected to externally controllable torque, introducing a torque ratio as a key control parameter. The adaptive crashworthiness of twisting metamaterials based on gyroid structures is then demonstrated under both quasi‐static and dynamic compression, with direct comparison to primitive (untwisted) gyroid counterparts.^[^ ^40^ ^]^ Three torsional boundary conditions were imposed—locking, free‐to‐twist, and over‐rotated—so that the experimental setup enabled simultaneous measurement of axial (force–displacement) and torsional (torque–rotation) responses, even under dynamic loading. Both primitive and twisting gyroid structures were additively manufactured via powder bed fusion with 10% relative density using FE7131 steel. To extend understanding beyond this density, a parametric finite element study was conducted across 5–15% relative densities. Validation of the macroscale model required constitutive characterization of the parent material; therefore, bulk FE7131 properties were determined under quasi‐static and dynamic conditions, modelled using the Johnson–Cook constitutive framework, and supported by microstructural, microhardness, and crystallographic analyses of both bulk and gyroid‐sheet specimens. For accurate numerical–experimental alignment, geometric imperfections introduced by additive manufacturing were quantified by comparing CAD geometries with micro‐CT reconstructions of the printed structures. Finally, the adaptive energy absorption performance of the twisting gyroid was mapped onto an Ashby chart and generalized through an ad hoc predictive scaling law, thereby extending classical cellular materials theory to incorporate adaptive effects.

## 2 Mechanics of Twisting Metamaterials

### 2.1 Cosserat Continuum Mechanics

Cosserat continuum theory, also known as the micropolar continuum, extends classical Cauchy elasticity by introducing independent rotational degrees of freedom at each material point, enabling a more complete representation of material behavior, including couple stresses and microstructural effects.^[^ ^36^ ^]^ Cosserat theory introduces the microrotation field in addition to the displacement one, defining the micropolar strain tensor (ε_*ij*_) and curvature tensor (*k_ij_*). These tensors are usually expressed component‐wise using Eringen's notation^[^ ^41^ ^]^ as follows.

(1) $$\;\varepsilon_{ij}=\frac{\partial u_{j}}{\partial x_{i}}+\in_{jik}\phi_{k}$$

(2) $$\;k_{ij}=\frac{\partial \phi_{j}}{\partial x_{i}}\;$$

where ϕ_*i*_ and *u_i_* are the components of the microrotation and displacement fields, respectively, while ∈_*jik*_ represents the Levi‐Civita tensor. Cosserat continuum theory introduces two distinct stress tensors: the force stress tensor (σ_*ij*_), analogous to the classical Cauchy stress tensor, and the couple stress tensor (*m_ij_*) to account for local moment effects. The constitutive equation for the force and couple stresses can be derived from the strain energy density. For a physically linear micropolar‐elastic material,^[^ ^42^ ^]^ the strain energy density (L) in elastic regime takes a quadratic form in terms of the micropolar strain and curvature tensors:

(3) $$L=\frac{1}{2}\varepsilon_{ij}C_{ijkl}\varepsilon_{kl}+\varepsilon_{ij}D_{ijkl}k_{kl}+\frac{1}{2}k_{ij}A_{ijkl}k_{kl}$$

where *A_ijkl_* and *C_ijkl_* are 4th order elasticity tensors with symmetry properties: *C_ijkl_* = *C_klij_*  and *A_ijkl_* = *A_klij_* . *D_ijkl_* is a rank‐four pseudo‐tensor that reverses its sign under space inversion, responsible for coupling strain and curvature, and capable of predicting material handedness due to architecture chirality.^[^ ^37^ ^]^ The energy density in Equation (3) contains 171 independent material parameters since both ε_*ij*_ and *k_ij_* are generally non‐symmetric tensors. However, material symmetries, such as isotropy, greatly reduce the number of independent elastic constants. For instance, an isotropic micropolar‐elastic material requires only six independent elastic constants.^[^ ^42^ ^]^

The constitutive equations for the force stress tensor (σ_*ij*_) and couple stress tensor (*m_ij_*) are derived by differentiating the strain energy density (L) in Equation (3) with respect to micropolar strain and curvature tensors:

(4) $$\sigma_{ij}=\frac{\partial L}{\partial \varepsilon_{ij}}\;=C_{ijkl}\;\varepsilon_{kl}+D_{ijkl}k_{kl}$$

(5) $$\;m_{ij}=\frac{\partial L}{\partial k_{ij}}=A_{ijkl}\;k_{kl}+D_{klij}\varepsilon_{kl}$$

In the special case, where *C_ijkl_* = *C_klij_*  = *C_jikl_*  and the coupling tensors vanish, i.e., *D_ijkl_* = *A_ijkl_*  =  0, the Cosserat continuum theory reduces to the traditional Cauchy continuum theory. Therefore, the Cosserat theory offers a generalized continuum framework capable of capturing critical material features where traditional Cauchy theory falls short, such as material size‐dependency,^[^ ^36^ ^]^ chirality,^[^ ^43^ ^]^ and anisotropy.^[^ ^44^ ^]^ Cosserat theory can also pave the way for the development of innovative materials with advanced mechanical properties, as illustrated by this study's focus on adaptive energy‐absorbing materials.

### 2.2 Energy Absorption Capability of Twisting Metamaterial

The crashworthiness of materials is typically assessed under uniaxial compression because it is directly linked to the force transmitted to the component to protect and the amount of energy dissipated during an impact load. This implies focusing on key mechanical properties such as axial stiffness, initial collapse stress, and specific energy absorption. A twisting metamaterial exhibits force stress coupled with curvature under compression load, exhibiting the behavior of a micropolar‐elastic material within the Cosserat continuum framework. To simplify the notation in uniaxial compression, repeated indices were replaced with a single subscript “1”, which corresponds to the loading direction, simplifying the expressions for force stress (σ_1_) and couple stress (*m*_1_) as follows: σ_1_ = *C*_1_ ε_1_ + *D*_1_ *k*_1_ and *m*_1_ = *A*_1_ *k*_1_ + *D*_1_ ε_1_. Consequently, the force stress of micropolar‐elastic material was rearranged from Equation (4) by incorporating the couple stress from Equation (5) as follows:

(6) $$\sigma_{1}=C_{1}\;\varepsilon_{1}+\frac{D_{1}}{A_{1}}\left(m_{1}-D_{1}\varepsilon_{1}\right)$$

The Equation (6) shows that the force stress depends on both constitutive parameters *C*_1_,*A*_1_, and *D*_1_ and couple stress aligned with the axial direction (*m*_1_): a controllable external torque (*M*) applied over the micropolar‐elastic material cross‐section (*A*_0_). In the linear elastic regime, the external torque (*M*) applied to the material can be linearly approximated as follows:

(7) $$\;m_{1}=\frac{M}{A_{0}}=\eta \varepsilon_{1}$$

where η determines how much torque is exerted to the material during the axial deformation. Therefore, the force stress in Equation (6) was rewritten using Equation (7) as follows:

(8) $$\;\sigma_{1}=\left[C_{1}+\frac{D_{1}}{A_{1}}\left(\eta -D_{1}\right)\right]\;\varepsilon_{1}$$

Interestingly, preventing the micropolar‐elastic material rotations during axial compression means the curvature (*k*_1_) becomes zero because of an external torque (*M_L_*) which is not constant, as inferable by Equation (5):

(9) $$\frac{M_{L}}{A_{0}}=\;\eta \;\varepsilon_{1}=D_{1}\;\varepsilon_{1}$$

**Figure 1:**
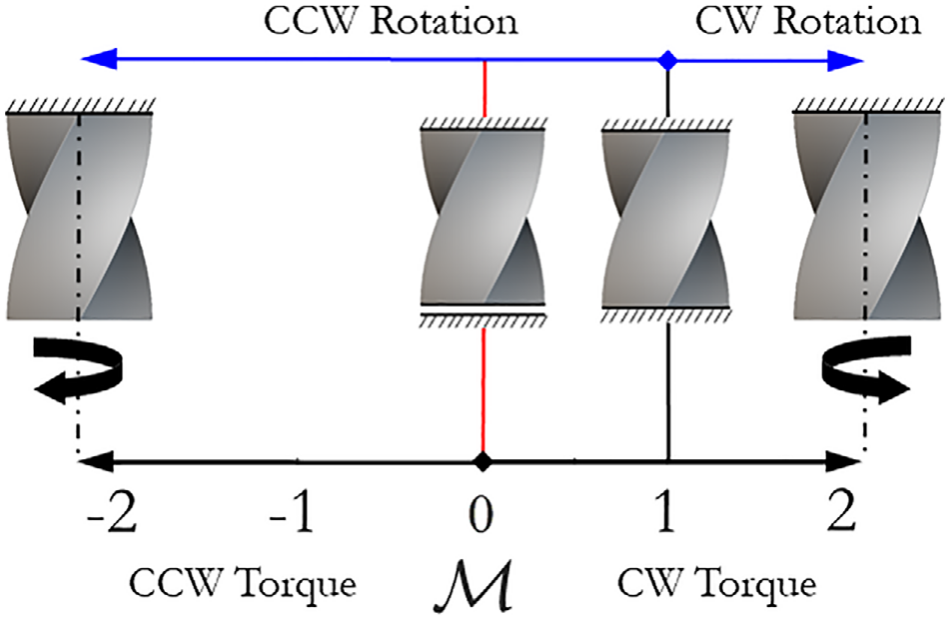
Influence of torque ratio (M) on the torque directions, rotations, and torsional loading configurations of CCW twisting metamaterial (right‐handed micropolar metamaterial).

That specific boundary condition allows introducing the dimensionless parameter $M=\frac{M}{M_{L}}=\frac{\eta }{D_{1}}\;$, called torque ratio, capable of identifying the torsional configurations induced by external torques. For a better understanding, **Figure** **1** summarizes how variations in the torque ratio affect the torque directions, rotations, and torsional loading configurations in counterclockwise (CCW) twisting metamaterials: right‐handed micropolar metamaterial (positive *C*_1_,*A*_1_, and *D*_1_ constitutive parameters).

Notably, for M<0 the twisting metamaterial experiences over‐rotation due to an external positive torque (driving torque) aligned with the twisting direction (CCW). On the contrary, for M>1, the twisting metamaterial experiences over‐rotation in the opposite direction, clockwise (CW) direction, caused by an external negative torque (driving torque). 0<M<1 is a peculiar range of twisting metamaterial delimitated by two specific torsional configurations: locking (M=1) and free‐to‐twist (M=0) conditions. The external torque within the range 0<M<1 counters the material rotation induced by the geometric twisting. As a result, the twisting metamaterial experiences under‐rotation in the twisting direction (CCW), with the torque reversing direction and acting as a resistive torque. It is noteworthy to highlight that the range 0<M<1 is not exhibited by cellular materials governed by classical Cauchy continuum mechanics because the locking (M=1) and free‐to‐twist (M=0) conditions coincide. Additional details based on strain energy density were provided in Section S1 (Supporting Information).

Based on the torque ratio parameter, the Equation (8) was further rearranged to highlight the influence of external torque on the force stress of micropolar‐elastic material:

(10) $$\sigma_{1}=\left[C_{1}+\frac{{D_{1}}^{2}}{A_{1}}\left(M-1\right)\right]\;\varepsilon_{1}$$

Thus, the force stress defined in Equation (10) was substituted into the strain energy density expression, Equation (3), obtaining the micropolar‐elastic energy density for uniaxial compression in elastic regime:

(11) $$L=\frac{1}{2}\left[C_{1}+\frac{{D_{1}}^{2}}{A_{1}}\left(M^{2}-1\right)\right]\;{\varepsilon_{1}}^{2}=\frac{A_{1}}{2}\left[\frac{C_{1}A_{1}+{D_{1}}^{2}\left(M^{2}-1\right)}{{\left(C_{1}A_{1}+{D_{1}}^{2}\left(M-1\right)\right)}^{2}}\right]{\sigma_{1}}^{2}$$

If micropolar‐elastic materials were twisting metamaterial designed by applying a twisting operation on the primitive architected materials, they behave like primitive one under compression, exhibiting the characteristic compressive response composed by elastic regime, flat plateau in post‐collapse regime, and densification. Therefore, the adaptive energy absorption capability of twisting metamaterial can be inferred by evaluating the initial collapse stress (σ_*P*_) of micropolar‐elastic material because the densification strain (ε_*d*_) is only dependent on material relative density. Assuming the initial collapse stress (σ_*p*_) occurs once the material reaches a critical energy density value ($L_{c}$) in elastic regime, σ_*p*_ can be expressed as function of torque ratio (M) using Equation (11) as follows:

(12) $$L_{c}=\;\frac{A_{1}}{2}\left[\frac{C_{1}A_{1}+{D_{1}}^{2}\left(M^{2}-1\right)}{{\left(C_{1}A_{1}+{D_{1}}^{2}\left(M-1\right)\right)}^{2}}\right]{\sigma_{p}}^{2}$$

By selecting the locking condition (M=1) as the reference initial collapse stress (σ_*PL*_), the influence of external torsional loading on the σ_*p*_ of micropolar‐elastic materials was derived from Equation (12) as follows:

(13) $$\frac{\sigma_{p}}{\sigma_{PL}}=\sqrt{\frac{{\left(C_{1}A_{1}+{D_{1}}^{2}\left(M-1\right)\right)}^{2}}{\;C_{1}A_{1}\left(C_{1}A_{1}+{D_{1}}^{2}\left(M^{2}-1\right)\right)}}\;$$

$\frac{\sigma_{P}}{\sigma_{PL}}$ reaches the maximum values when M=1 and decreases applying a driving torque (M>1,M<0) or a resistive torque 0<M<1 to the material.

**Figure 2:**
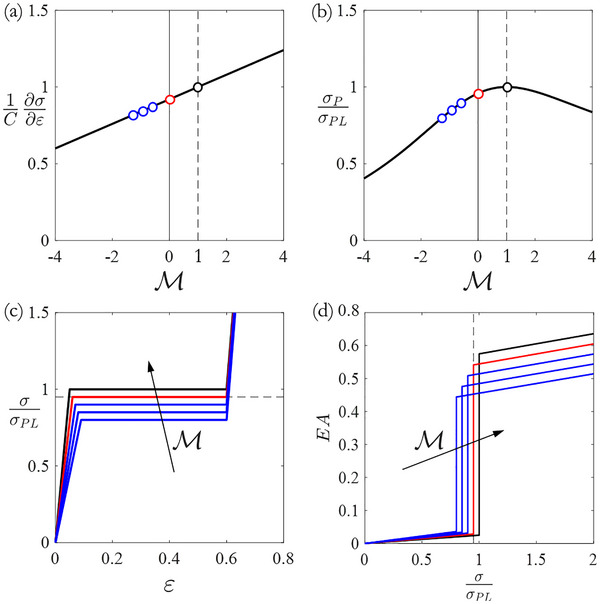
Influence of torque ratio (M) on the mechanical properties and energy absorption capability of CCW twisting metamaterial (right‐handed micropolar metamaterial). a) Axial stiffness. b) Initial collapse stress. c) Axial stress‐strain curve. d) Energy absorption capability. Black, red, and blue dots and lines correspond to different torsional loading configurations: M=1, M=0, and M<0, respectively.

**Figure** **2** summarizes the torque ratio (M) influences on axial stiffness, initial collapse stress, and energy absorption capability of micropolar‐elastic material, in which the mechanical performances are normalized to locking (M=1) condition. The axial stiffness, obtained from the Equation (10) as ∂σ_1_/∂ε_1_, linearly depends on the external torque applied during axial loadings, decreasing as the torque is aligned with the twisting direction, as shown in Figure 2a. The initial collapse stress reaches the maximum values at M=1, as show in Figure 2b, thus the characteristic compressive response exhibits a flat plateau in post‐collapse regime which reduces as torque ratio decreases, as illustrated in Figure 2c where the stress‐strain curves correspond to different torsional loading configurations: black for M=1, red for M=0, and blue for M<0.

Therefore, twisting metamaterials exhibit the highest initial collapse stress and energy absorption values when their rotations are fully restricted (M=1), while the application of an external resistive or driving torque results in decreasing energy absorption performances. The analytical description leads to the inference that the twisting metamaterials adapt their stiffness, collapse stress, and energy absorption capabilities in response to torsional constraints, actively controllable. Indeed, the stress‐strain curves can also be integrated to yield energy absorption diagram, Figure 2d, to visualize the absorption capacity area covered by the effect of torque ratio on normalized energy absorption capacity $EA=\frac{1}{\sigma_{PL}}\int_{0}^{\varepsilon }\sigma \;d\varepsilon$. A peculiar advantage of twisting metamaterials lies in the operational range 0<M<1. From an engineering perspective, the endpoints of this range can be practically realized using a rotational base located between the material and the component to protect. Equipping the rotational base with locking (M=1) and unlocking (M=0) rotations device, these two configurations can be readily implemented. Furthermore, it is worth noting that operating within the 0<M<1 regime necessitates the application of a resistive torque, which can be induced through braking mechanisms or rotational inertia devices, such as flywheels. Therefore, twisting metamaterials offer new functional designs for energy harvesting applications where impact energy can be partially converted into rotational kinetic energy, stored and, later, released through torsional motion.

## 3 Results and Discussion

### 3.1 Mechanical Response of Twisting Gyroid Structures

#### 3.1.1 Quasi‐Static Compression

**Figure 3:**
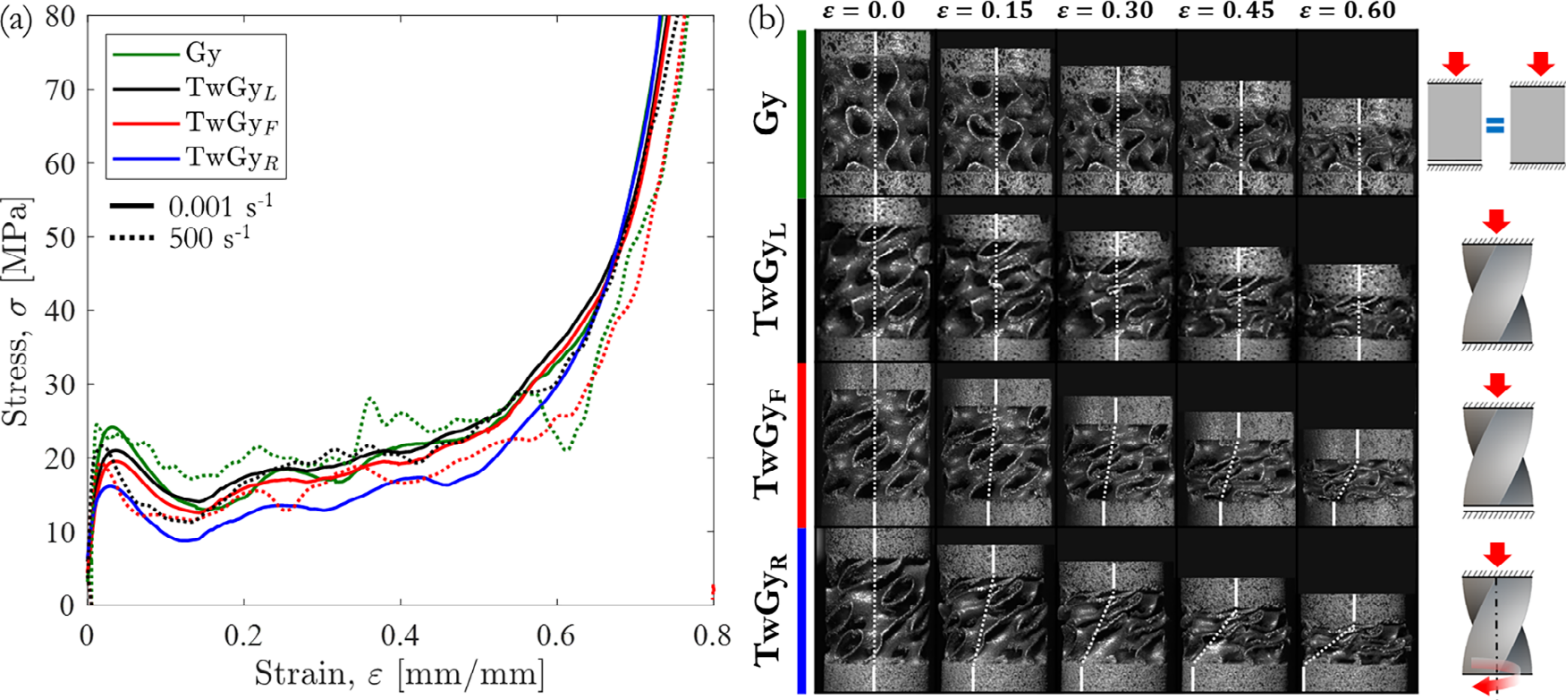
Quasi‐static and dynamic compression behavior of gyroid structures with $\bar{\rho }$= 10%. a) Characteristic engineering stress‐strain responses and b) deformation maps at various stages, including collapse modes, rotations, and torsional constraints. The torsional constraints are defined by “TwGy_F_” (M=0), “TwGy_L_” (M=1), and “TwGy_R_” (M=−1) loading configurations.

The compressive response of both primitive and twisting gyroid structures with a relative density $\bar{\rho }$ = 10% was investigated under quasi‐static loading. **Figure** **3** summarizes their corresponding macroscopic compressive stress–strain responses, deformation and failure modes, captured at three distinct torsional boundary conditions: locking condition “TwGy_L_” (M=1), free‐to‐twist condition “TwGy_F_” (M=0), and rotative condition “TwGy_R_” (M=−1). Each stress–strain curve for the gyroid structures exhibited an initial linear‐elastic region, followed by a sudden stress drop, a plateau regime characterized by post‐collapse stress oscillations, and a final densification phase. The collapse mechanism was dominated by yielding and widespread plastic deformation, leading to the formation of macroscopic crush bands. Following the first crush band (i.e., the initial collapse stress), the ductility of the gyroid‐sheet material enabled a stable compressive response without catastrophic failure, resulting in a progressive, band‐by‐band collapse approaching full densification (see Videos S2 and S3, Supporting Information).

The rotational behavior of the twisting gyroid structures is illustrated in Figure 3b by connecting a dotted white line between two reference points located at the upper and lower boundaries of the structures. During compression, this line inclines, visually indicating the degree of rotation in the vertical plane. It remains vertical when no rotation occurs—either due to constrained motion, as in the “TwGy_L_” (M=1) configuration, or due to geometric restrictions, as in the “Gy” configuration. As the inclination increases, it reflects enhanced rotation, most notably in the “TwGy_F_” (M=0) configuration (ϕ≅28° at ε  =  0.6), and even more significantly in the “TwGy_R_” (M=−1) configuration (ϕ≅90° at ε  =  0.6).

The compressive mechanical response of the twisting gyroid structures was strongly influenced by the applied torsional boundary conditions, as illustrated by stress–strain curves in Figure 3a. By evaluating the slope of the initial elastic regime (defined as  *E_p_* = ∂σ_1_/∂ε_1_ ), it is evident that the axial stiffness of “TwGy” structures declines as the torque ratio M decreases. Specifically, the “TwGy_L_” (M=1) configuration exhibited the highest axial stiffness (4.8 GPa), while this value dropped to 4.1 GPa under free‐to‐rotate conditions (“TwGyF”, M=0), and further to 3.6 GPa in the over‐constrained counterclockwise case (“TwGyR”, M=−1). These elastic responses under various torsional constraints were accurately modeled using a mesoscale micropolar‐elastic material formulation grounded in Cosserat continuum mechanics. The loading cases used for calibrating the constitute parameters are summarized in Table S1 (Supporting Information), employing Equations (4) and (5). From the experimental results, the elastic constants were determined as:  *C*_1_ =  4812 MPa, *D*_1_ =  2.11 N/mm, and *A*_1_ =  0.0074 N/rad. All constants were positive, confirming that the twisting gyroid behaves as a right‐handed micropolar metamaterial.

During the plateau regime, the initial collapse stresses (σ_*p*_) and post‐collapse stress fluctuations were also affected by the torsional boundary conditions, even if the collapse mechanism was the same: crush bands triggered by local yielding. Both σ_*p*_ and post‐collapse stress plateau of “TwGy” structure reduces as the torque ratio (M) reduces. Notably, the σ_*p*_ decreases from a maximum value of 21 MPa in “TwGy_L_” configuration to a minimum of 16 MPa in “TwGy_R_” configuration. The theoretical collapse stress values can be defined by Cosserat continuum theory using Equation (13) and the already calibrated constitutive parameters *C*_1_, *D*_1_, and *A*_1_.

**Table** **1** shows that the theoretical values are consistent with experimental ones, confirming that the mechanical properties of twisting gyroid structure under different torsional loading (−1<M<1) can be effectively predicted with the mesoscale micropolar‐elastic material model in Cosserat continuum mechanics. The energy absorption performance was experimentally evaluated by comparing the onset of densification strain (ε_*d*_), the initial collapse stresses (σ_*p*_), the energy absorption capacity ($W=\int_{0}^{\varepsilon_{d}}\sigma \;d\varepsilon$), and the specific energy absorption ($SEA=\frac{W}{\rho }\;$) where ρ is the average density of the unit‐cell of gyroid structures. The SEA computed at the onset of densification, identified with the maximum efficiency procedure, takes the name SEA_d_. Table 1 shows that the SEA_d_ of twisting gyroid structure with $\bar{\rho }=10\%$ reduces as the torque ratio reduces. The “TwGy” structure exhibited a maximum value of 15.36 J/g in “TwGy_L_” configuration and decreased up to 10.32 J/g in “TwGy_R_” configuration. Therefore, “TwGy” demonstrates adaptive energy absorption properties because of external torsional conditions. Assuming the free‐to‐twist condition (M=0) as neutral position, the “TwGy” structure changes SEA_d_ from ‐26.2% to +9.3% into the operation range −1<M<1, respectively.

**Table 1:** Theoretical, experimental, and numerical values of quasi‐static mechanical and energy absorption performances of gyroid structures.

	Sample Code	Unit cell topology	M[−]	*E_p_* [GPa]	σ_*p*_ [MPa]	ε_*d*_ [mm mm^−1^]	SEA_d_ [J g^−1^]
Exp.	Gy	Gyroid	1÷0	5.4 ± 0.28	24.22 ± 0.21	0.54 ± 0.01	15.41 ± 0.32
	TwGy_L_	Twist Gy.	1	4.8 ± 0.30	21.02 ± 0.25	0.55 ± 0.01	15.36 ± 0.35
	TwGy_F_	Twist Gy.	0	4.1 ± 0.31	19.52 ± 0.31	0.54 ± 0.01	14.05 ± 0.29
	TwGy_R_	Twist Gy.	−1	3.6 ± 0.33	16.25 ± 0.28	0.53 ± 0.01	10.32 ± 0.23
FEA	Gy	Gyroid	1÷0	5.5	24.05	0.61	15.71
	TwGy_L_	Twist Gy.	1	4.9	21.80	0.61	15.64
	TwGy_F_	Twist Gy.	0	4.2	19.85	0.60	14.46
	TwGy_R_	Twist Gy.	−1	3.7	16.53	0.59	11.01
Theory	Gy	Gyroid	1÷0	–	–	–	–
	TwGy_L_	Twist Gy.	1	4.8^a^ ^)^	21.02^b^ ^)^	–	15.41^c^ ^)^
	TwGy_F_	Twist Gy.	0	4.2^a^ ^)^	19.65^b^ ^)^	–	14.21^c^ ^)^
	TwGy_R_	Twist Gy.	−1	3.6^a^ ^)^	16.60^b^ ^)^	–	10.57^c^ ^)^

^a)^ Equation (10) with *C*_1_ =  4812 MPa, *D*_1_ =  2.11 N/mm, and *A*_1_ =  0.0074 N/rad;

^b)^ Equation (13) with *C*_1_ =  4812 MPa, *D*_1_ =  2.11 N/mm, *A*_1_ =  0.0074 N/rad, and σ_*PL*_ =  21.02 MPa;

^c)^ Equation (15) with *C*  =  1525 MJ/m^3^, α  =  2.10, and β  =   − 121 MJ/m^3^.

Comparing the primitive and twisting gyroid structure mechanical properties (Table 1), the “Gy” structure shows high stiffness and initial collapse stress compared to “TwGy”, whereas the energy absorption capabilities were comparable in “TwGy_L_” configuration (M=1). That happens because the post‐collapse stresses of both structures were similar, as well as the onset of densification strains, as shown in Figure 3, inferring that ε_*d*_ is only function of relative density. Decreasing the torque ratio, the ε_*d*_ slightly decreases as shown in Table 1.

**Figure 4:**
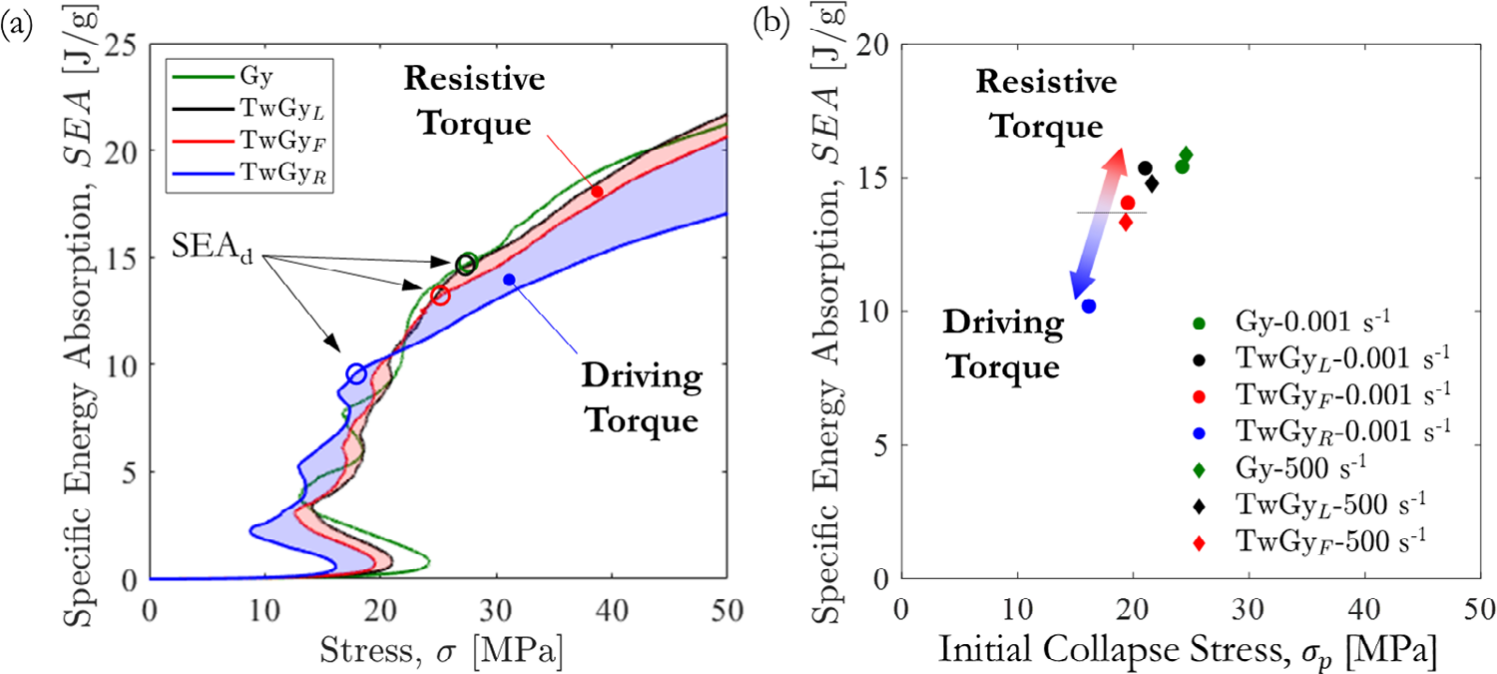
Energy absorption diagrams of primitive and twisting gyroid structures with relative density $\bar{\rho }=10\%$. a) Effect of external torque on energy absorption under quasi‐static loading. b) Specific energy absorption at the onset of densification (SEA_d_) measured under quasi‐static and dynamic conditions. Torsional boundary conditions are defined as “TwGy_F_” (M=0), “TwGy_L_” (M=1), and “TwGy_R_” (M=−1).

Further analysis through energy absorption diagrams (see **Figure** **4**) revealed that the “TwGy” structure displayed a shaded region representing attainable SEA values within the operational torque range. The blue‐shaded region corresponds to scenarios with externally applied driving torque, while the red‐shaded region reflects SEA under resistive torque. This visual clearly demonstrates the twisting metamaterial's capability to modulate both force transmission and energy dissipation under impact loads. In high‐energy dissipation applications, the structure should be torsionally fixed to maximize absorption, while in load‐limiting applications, free rotation can be permitted to reduce transmitted force. It is noteworthy that external torque can also be applied to primitive gyroid structures, generating a corresponding shaded SEA region in Figure 4a. However, in this case, only the blue‐shaded region applies, as the free‐to‐twist and locked states coincide due to structural symmetry. The red‐shaded region is a distinguishing feature exclusive to twisting gyroid structures—or more broadly, to twisting metamaterials.

#### 3.1.2 Dynamic Compression

The compression behavior of both primitive and twisting gyroid structures with two different torsional boundary conditions was evaluated at high strain rates. The macroscopic stress–strain responses of gyroid structures with a relative density of $\bar{\rho }$ = 10% at an engineering strain rate of 500 s^−1^ are shown in Figure 3a. These dynamic stress–strain curves closely follow the quasi‐static behavior, with a slightly more pronounced stress drop post‐collapse. The strain rate sensitivity of the parent material did not significantly affect the initial collapse stress of the gyroid structures. Notably, the twisting gyroid structure continued to rotate freely under high‐speed impact conditions (≈6 m s^−1^). Experimental results confirm that the distinctive rotational behavior of twisting metamaterials is retained under dynamic loading (see Video S1, Supporting Information). The SEA_d_ values measured under dynamic loading are consistent with those obtained under quasi‐static conditions, as illustrated in Figure 4b. This confirms that the adaptive energy absorption behavior of the twisting gyroid structure is preserved at high strain rates. Figure 4b also highlights that the twisting gyroid structure in the “TwGy_L_” configuration exhibits a lower initial collapse stress than the “Gy” configuration, despite their comparable SEA_d_ values. Compared to primitive gyroid structure, the twisting metamaterial reduces the peak force transmitted to the protected component while maintaining equivalent energy absorption. This result demonstrates that incorporating geometric twisting into the design of architected materials can significantly enhance energy absorption performance and protective efficiency relative to untwisted structures.

### 3.2 Bulk and Gyroid‐Sheet Material Characterization

The quasi‐static and dynamic compression tests on the FE7131 bulk samples revealed high strength and ductility, as shown by the true stress‐strain curve in Figure S1 (Supporting Information). Under quasi‐static condition, the compressive yield stress, estimated as σ_0.2%_, was ≈780 MPa followed by strain hardening, which led to a stable compression stress of ≈1110 MPa at large deformation; no failures were exhibited. Increasing the strain rate up to 1000 s^−1^, the bulk material showed a moderate strain rate sensitivity, as σ_0.2%_ increased to 850 MPa. The Young's modulus was ≈195 GPa, determined via Digital Image Correlation (DIC) analysis. The mechanical behavior was implemented into Abaqus environment using the Johnson‐Cook material model; hence, strain rate sensitivity and thermal softening of the gyroid‐sheet material were accounted for in FE simulations of gyroid structures. The Johnson‐Cook model was calibrated following the procedure described in previous work;^[^ ^45^ ^]^ its parameters are listed in Table S2 (Supporting Information). The results of EDS microanalysis are reported in Table S3 (Supporting Information), confirming consistent chemical composition between bulk and gyroid‐sheet material. The XRD patterns in Figure S2 (Supporting Information) revealed peaks corresponding to the α‐Fe (ferrite) body‐centred cubic (bcc) phase (ICDD 65–4899) with nominal lattice parameter a = 2.8670 nm. Peak broadening analysis, summarized in Table S4 (Supporting Information), shows a slightly finer crystallite size in gyroid‐sheet material than bulk material, attributed to the faster cooling rates in PBF‐LB manufacturing of gyroid structure. Both lattice parameters were consistent with the nominal value (Δa/anom < 0.25%), demonstrating minimal crystallography variations and consistent fully ferritic microstructure of bulk and gyroid‐sheet material. XµCT analysis quantified the percent of closed porosity (CP) equal to 0.5% ± 0.1%, and 0.12 ± 0.04% were found for the bulk and gyroid‐sheet materials, respectively. However, even if the gyroid‐sheet material displayed a denser microstructure, both materials exceeded 99% relative density, demonstrating high structural integrity. The Vicker microhardness (HV) measurements in three different areas of gyroid‐sheet material (lines S1‐316HV, S2‐330HV, and S3‐324HV in Figure S3, Supporting Information) demonstrated homogeneity in mechanical response across the twisting gyroid structure. Despite the slight microstructural differences between gyroid‐sheet and bulk material due to the manufacturing process, their mechanical properties can be considered equivalent for the purposes of this study and homogeneously distributed, confirming the Johnson‐Cook model implementation into the macroscale model.

### 3.3 Predicting Mechanical Response of Twisting Gyroid Structures

#### 3.3.1 Effect of AM‐Induced Shape Variations

In additively manufactured architected materials, validating numerical models requires accurate information about the printed geometry, as the AM process typically introduces defects such as geometric and density variations, lack of fusion, internal porosity, trapped material, and other imperfections.^[^ ^46^ ^]^ Therefore, the numerical model of real 3D‐printed twisting gyroid was reconstructed from CT analysis and then used for a preliminary FE analysis.

**Figure 5:**
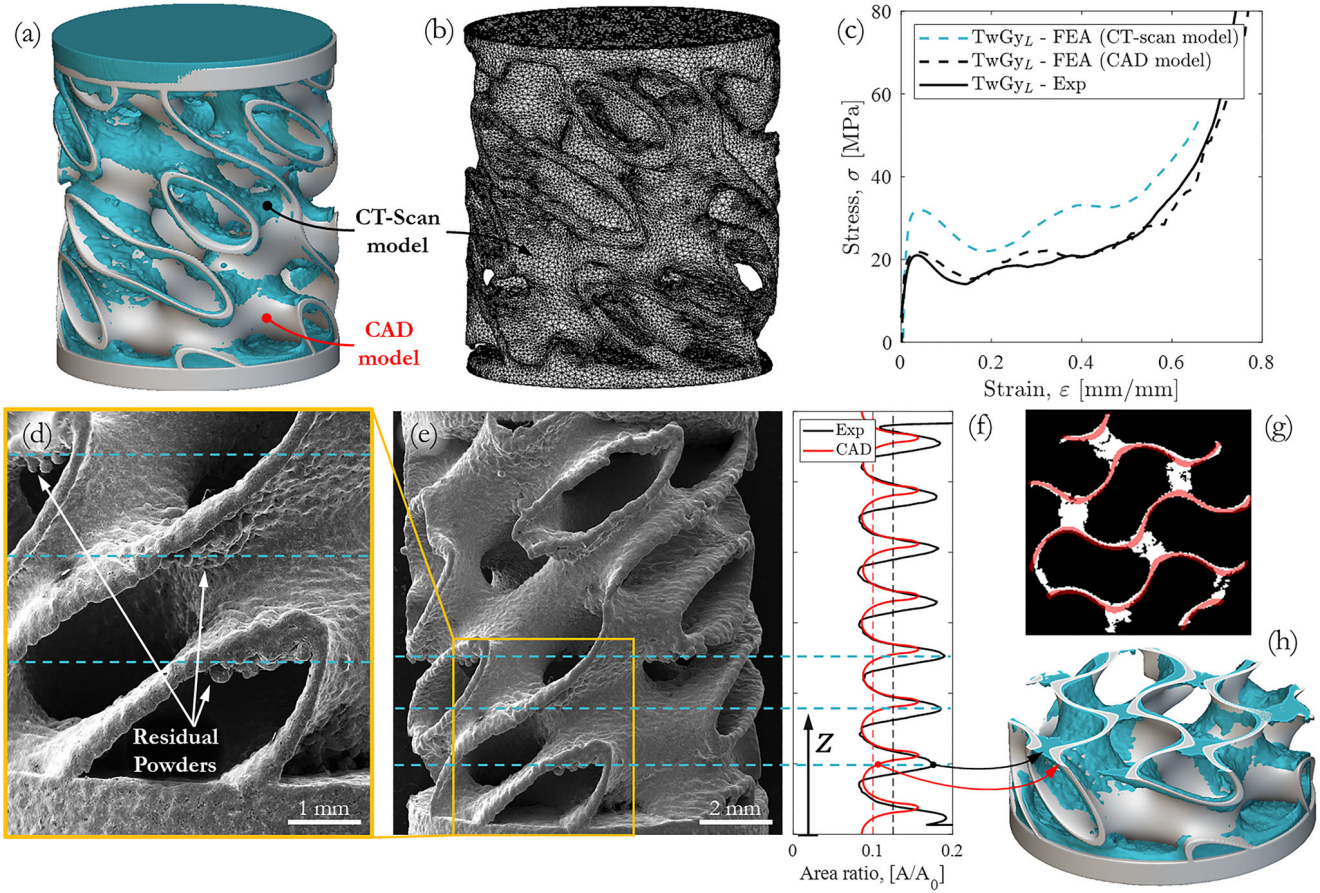
Effect of AM‐induced shape variations on the twisting gyroid structure. a) CT‐scan model superimposed on CAD geometry. b) Mesh generated from CT‐scan model. c) Experimental versus numerical compression response for the “TwGy_L_” (M=1) configuration. d,e) SEM images of gyroid wave surfaces. f) Area ratios along the build direction from CAD and CT‐scan models. g) Segmented layers of CAD and CT‐scan models. h) Comparison of CT and CAD geometry beneath a gyroid wave.

CT analysis of the 3D‐printed twisting gyroid structure revealed no porosity within the gyroid walls and only minor geometric deviations compared to the CAD model, as shown in **Figure** **5**a where the CT scan is overlaid over the CAD model. Figure 5a highlights excess material deposited along the gyroid waves down skins due to the additive manufacturing process, which altered the geometry and increased the relative density to ≈11.8%. Therefore, the CT‐scan model was meshed (Figure 5b) and used in preliminary FE simulations to account for the effect of the AM process on the effective geometry of the twisting gyroid structure in the “TwGy_L_” configuration. The stress‐strain responses for the CT‐scan and CAD models were numerically predicted. The CT‐based model exhibited a mechanical response 50% higher than the experimental results, as shown in Figure 5c, whereas CAD model closely aligned with the experimental data. This indicates that the CAD‐based predictions more accurately represent the actual mechanical response of the twisting gyroid structure, despite the CT‐scan model having a higher relative density because of manufacturing‐induced shape variations.

To investigate the underlying cause, a layer‐by‐layer comparison between the CT‐scan and CAD models was performed. Segmenting layers of both models, as shown in Figure 5, the cross‐sectional area (*A*) was measured and normalized by the reference area (*A*_0_). The resulting area ratios (*A*/*A*_0_) were plotted along the vertical direction of the gyroid structure (Z‐direction of the 3D printer) in Figure 5f, allowing the localization of excess material, as further illustrated by SEM images of gyroid waves down skins in Figure 5c,d. Significant differences in area ratios were observed beneath the gyroid wave connections, primarily due to residual powder adhering to inclined surfaces and critical unsupported regions, as illustrated in Figure 5h. The area ratios deviation can be attributed to manufacturing‐induced shape variations because of trapped metal powders beneath the gyroid wave connections, which are common imperfections in AM structures, known as overhang and bridging issues. In contrast, the area ratios above the gyroid wave connections matched between the CT‐scan and CAD models, indicating that the gyroid wall thickness remained consistent with the original design. As a consequence, the excess material observed beneath the connections was residual powder, which does not contribute to mechanical deformation, as supported by experimental/numerical findings from previous study^[^ ^47^ ^]^ and other researchers.^[^ ^48^, ^49^ ^]^ Thus, despite the higher relative density and minor shape variations in the CT‐scan model of 3D‐printed gyroid, the mechanical response of the twisting gyroid structure can be accurately predicted using the CAD model. This is because the structural regions of 3D printed gyroid are fully solid and geometrically consistent with the CAD model, whereas the excessive material (residual, partially sintered powders) merely increases the apparent density of the gyroid without contributing to its mechanical performance.

#### 3.3.2 FE Validation

**Figure 6:**
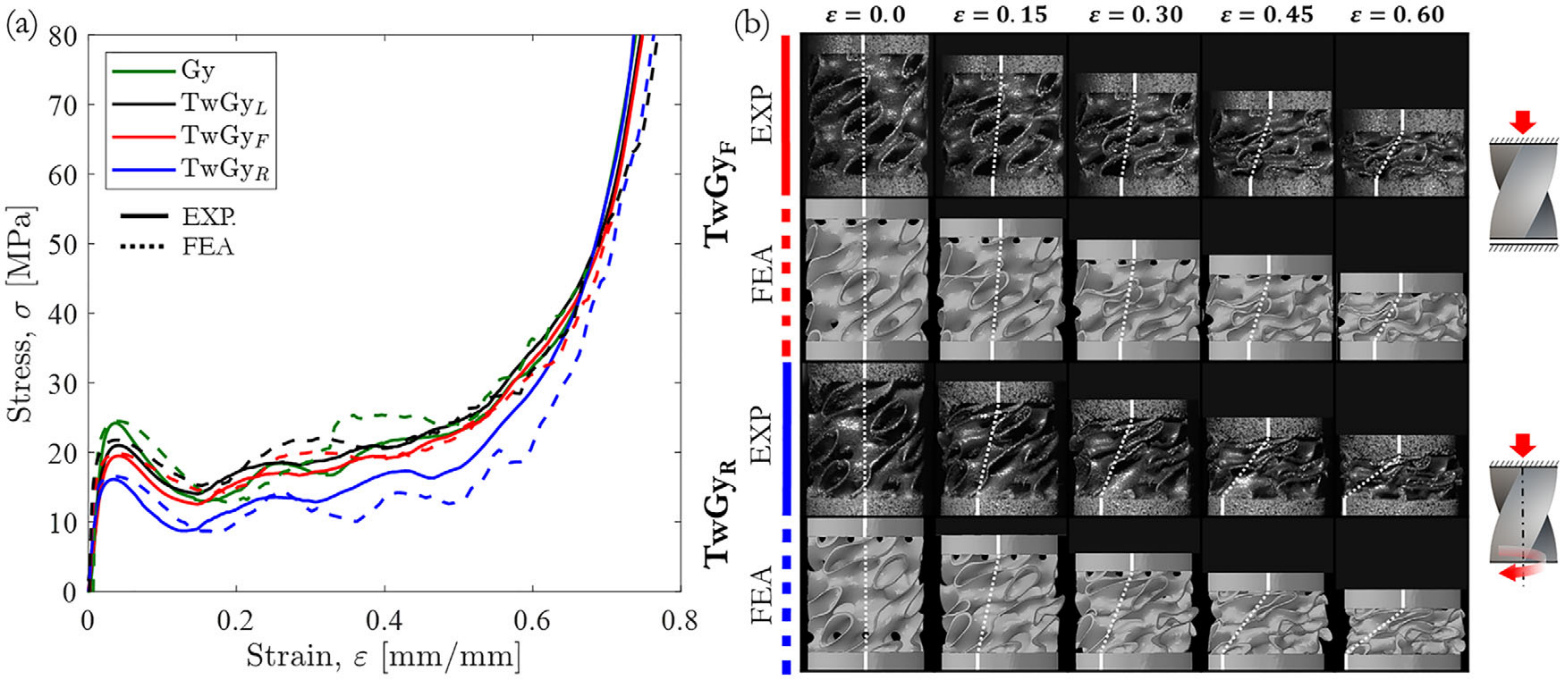
Experimental versus FE‐predicted quasi‐static compression behavior of gyroid structures with relative density $\bar{\rho }$= 10%. a) Engineering stress–strain responses. b) Deformation maps at different stages, highlighting collapse modes, rotations, and torsional constraints. Torsional boundary conditions are defined as “TwGy_F_” (M=0), “TwGy_L_” (M=1), and “TwGy_R_” (M=−1).

Using the CAD models, numerical predictions of both primitive and twisting gyroid structures ($\bar{\rho }=10\%$) with distinct torsional boundary conditions are compared with the experimental results in **Figure** **6** (see Videos S2 and S4, Supporting Information). The comparison shows characteristic engineering stress–strain curves, deformation maps, and rotations at various stages.

The numerical models effectively capture the compressive behavior of the gyroid structures, accurately predicting the onset of the initial crush band and the progressive post‐collapse response up to near‐complete densification. Additional details of collapse mechanism associated with the crush band are provided in Section S2 (Supporting Information). The predicted mechanical properties and energy absorption characteristics are summarized in Table 1, demonstrating the accuracy of the finite element (FE) model in predicting axial stiffness, initial collapse stress, and specific energy absorption (SEA_d_).

**Figure 7:**
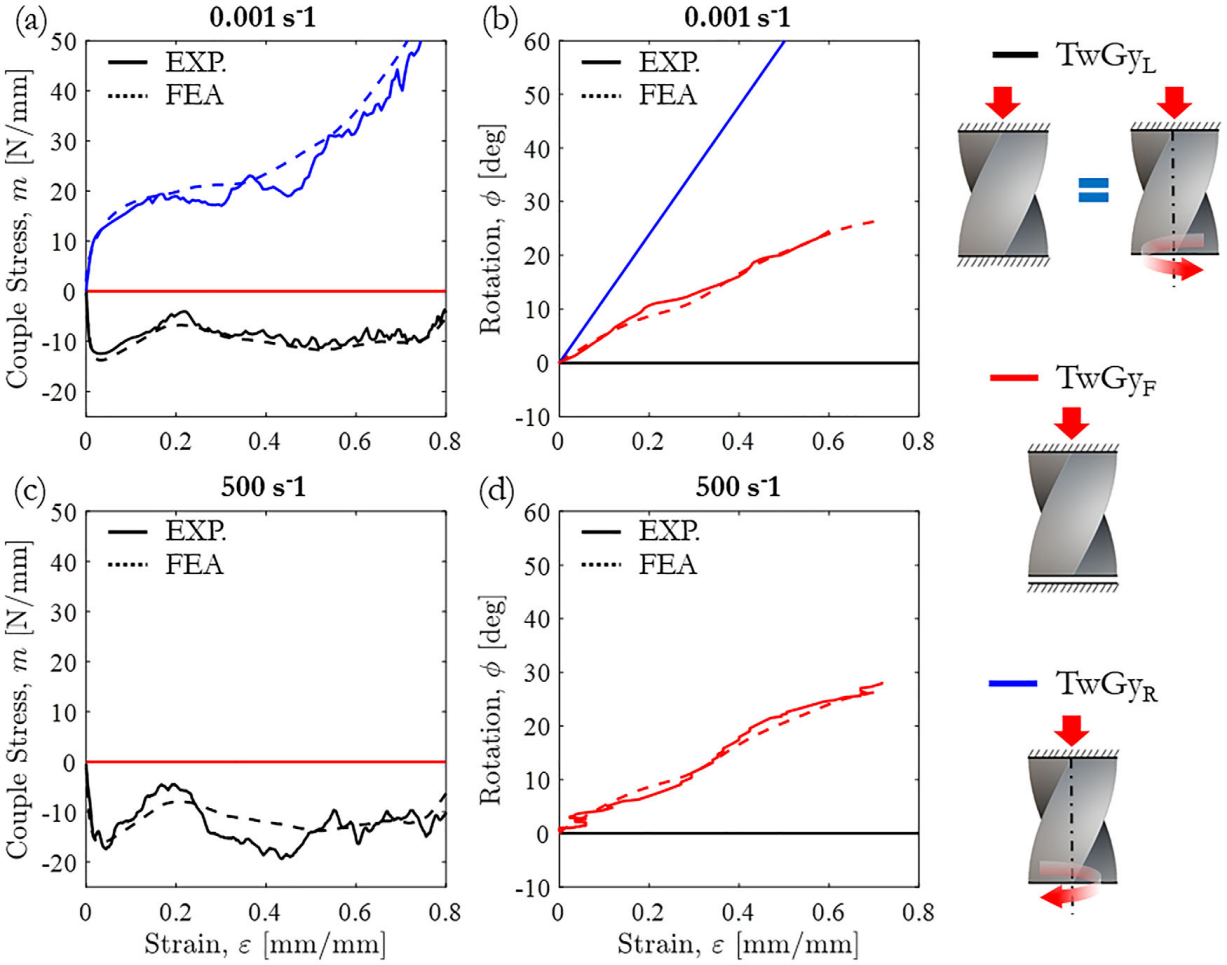
Experimental versus FE‐predicted rotational characteristics of the twisting gyroid structure with relative density $\bar{\rho }$= 10%. a) Quasi‐static couple stress–strain responses, and b) corresponding rotations. c) High strain rate couple stress–strain responses, and d) corresponding rotations. Torsional boundary conditions are defined as “TwGy_F_” (M=0), “TwGy_L_” (M=1) and “TwGy_R_” (M=−1).

The validation of the twisting gyroid structure's mechanical behavior using FE simulations extends beyond conventional stress–strain curves to include rotational responses (see Videos S2 and S4, Supporting Information). To evaluate this, experimental rotation angles and torques were compared with FE predictions. Figure 6b presents this comparison in the vertical plane (dotted white lines), showing strong agreement between simulations and experiments. For a deeper analysis, rotation (ϕ) and couple stress (*m*), as defined in Cosserat continuum mechanics, were plotted as functions of axial strain in **Figure** **7**, summarizing the quasi‐static and dynamic rotational responses of the twisting gyroid structure under three torsional boundary conditions: “TwGy_L_,” “TwGy_F_,” and “TwGy_R_”. Notably, the evolution of couple stress was accurately captured across both elastic and post‐collapse regimes.

Figure 7a shows a negative couple stress in the locked condition (M=1), where rotations are restricted by an externally applied clockwise (CW) torque. In contrast, the couple stress becomes positive when a counterclockwise (CCW) torque—aligned with the natural twisting direction of the metamaterial—is applied, inducing over‐rotation. The comparison between simulated and measured rotations is shown in Figure 7b. For the “TwGy_R_” configuration, rotation values are nearly superimposed, as rotations were externally imposed by a universal tensile/torsion machine. However, for the “TwGyF” configuration, slight discrepancies are observed between predicted and experimental rotations. This is because in “TwGy_F_” the rotation was geometrically induced by the structure's design, so influenced by contact interactions, geometric imperfections, and friction between crushed zones.

At high strain rates, the rotational response of the twisting gyroid structure remains consistent with that observed under quasi‐static loading, as shown in Figure 7d. This consistency indicates that the coupled torsional–axial motion is purely kinematic, governed solely by the geometry of the twisting metamaterial. However, the couple stress–strain response increases slightly, similar to the axial stress–strain behavior, due to the strain‐rate sensitivity of the gyroid‐sheet material, Figure 7c. This implies that while axial and torsional stresses at high strain rates are governed by material properties, the deformation mechanisms and axial–rotational coupling are dictated by the architecture of the twisting metamaterial. The comprehensive comparisons presented in Figure 7 and Figure S4 (Supporting Information), along with the supplementary videos, confirm the predictive capability of the FE model across both axial and rotational behaviors.

### 3.4 Energy Absorption Capability of Twisting Metamaterials

**Figure 8:**
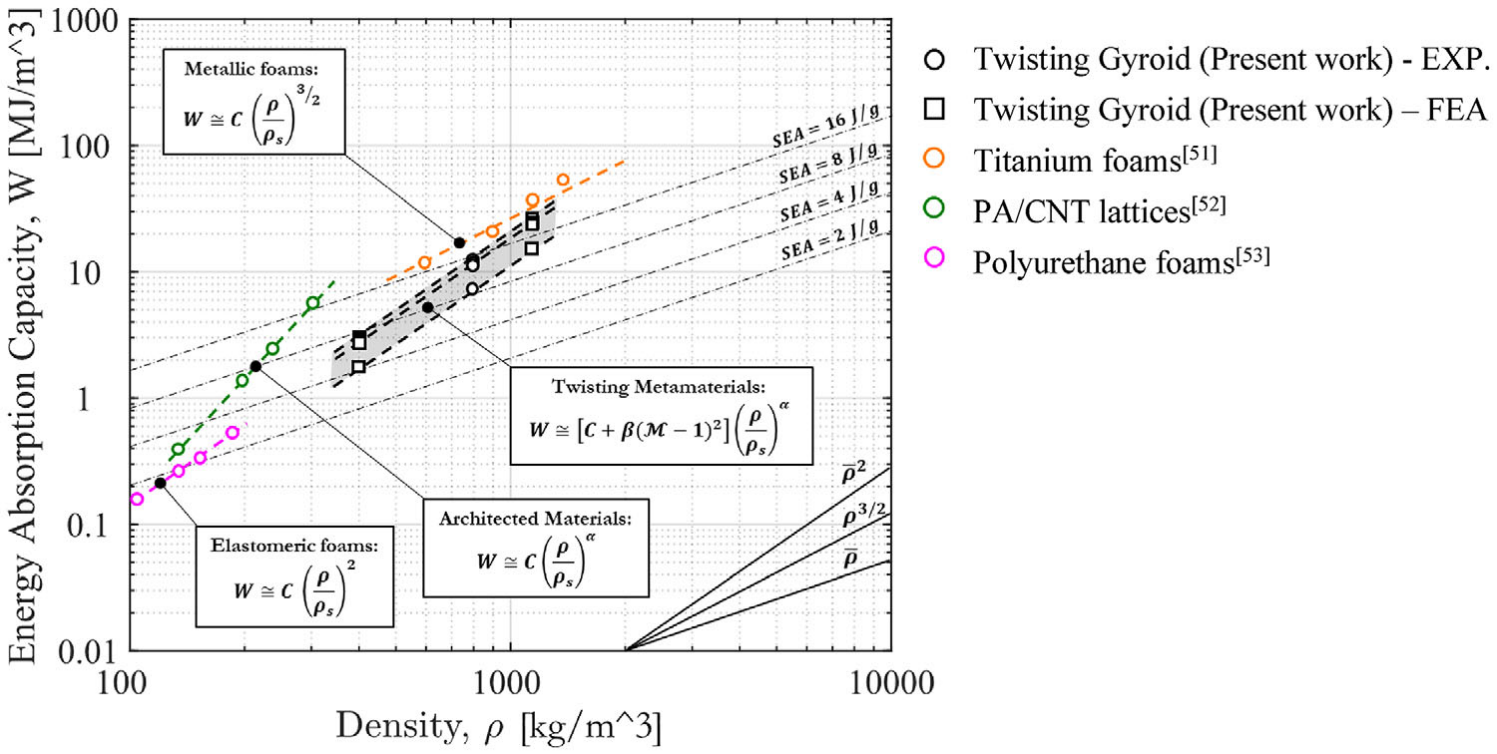
Adaptive energy absorption capability of twisting metamaterials on Ashby chart.

The previous sections explored the numerical and experimental mechanical properties of gyroid structures, emphasizing the energy absorption characteristics of twisting gyroid structures with $\bar{\rho }$ = 10% based on torsional boundary conditions. In addition to macroscale and mesoscale predictive models, the mechanical properties of the twisting gyroid structure can also be predicted using scaling laws, following the mechanics of the cellular material approach.^[^ ^50^ ^]^ For instance, the energy absorption capacity of cellular materials can be expressed as:

(14) $$W=C{\left(\frac{\rho }{\rho_{s}}\right)}^{\alpha }$$

where ρ_*s*_ is bulk material density. *C* depends both base material and topology, whereas α is topology‐dependent constant; α typically ranges from 1.5 (plastic foams) to 2 (elastomeric foams), while 3D lattices (architected materials) can exhibit values up to 3 based on the architectures. This relationship is typically represented as a single curve on an Ashby chart because traditional cellular materials exhibit fixed energy absorption at a given relative density. For twisting gyroid structures, which exhibit adaptive energy absorption, Equation 14 is insufficient. Since the densification strain ε_*d*_ is unaffected by the torque ratio, the energy absorption scales with the initial collapse stress: $W\left(M\right)\;∼\;\sigma_{p}\left(M\right)\varepsilon_{d}$, with $C=W\left(1\right)\;∼\;\sigma_{PL}\varepsilon_{d}$ for the locking condition (M=1). Incorporating the torque ratio effect, the scaling law can be updated as:

(15) $$W\;\left(M\right)=C\frac{\sigma_{p}}{\sigma_{PL}}{\left(\frac{\rho }{\rho_{s}}\right)}^{\alpha }≅\left[C+\beta {\left(M-1\right)}^{2}\right]{\left(\frac{\rho }{\rho_{s}}\right)}^{\alpha }$$

where β is a topology‐dependent constant capturing the torque ratio influence, approximating Equation 13 with a quadratic form. Equation 15 enables theoretical predictions of the adaptive energy absorption capacity based on relative density and torque ratio. The formulation can be generalized to other adaptive stimuli such as temperature, moisture, electric fields, or jamming. A parametric FE study explored twisting gyroid structures with $\bar{\rho }$ = 5% and $\bar{\rho }$ = 15%, and results are summarized in Table S5 (Supporting Information). On an Ashby chart (**Figure** **8**), the adaptive energy absorption capability of twisting metamaterials is represented as a shaded area rather than a single curve, compared with titanium foams,^[^ ^51^ ^]^ PA/CNT lattices,^[^ ^52^ ^]^ and polyurethane foams.^[^ ^53^ ^]^ This approach highlights the adaptive response as a unique property of twisting metamaterials. Twisting metamaterials can be derived from generic primitive lattices, establishing a general design strategy for adaptive energy‐absorbing architectures. The calibrated scaling law parameters for the present twisting gyroid are: *C*  =  1525 MJ m^−3^, α  =  2.10, and β  =   − 121 MJ m^−3^.

## 4 Conclusion

This study introduces twisting metamaterials as a new subclass of micropolar materials and establishes them as adaptive crashworthy materials under varying torsional constraints through theoretical formulations, multiscale predictive modeling, and experimental validation. The adaptivity concept was initially described using Cosserat continuum mechanics, modeling twisting metamaterials as micropolar‐elastic materials subjected to a controllable external torque, with the torque ratio (M) serving as a torsional control parameter. Theory demonstrates that twisting metamaterials can modulate axial stiffness, initial collapse stress, and energy absorption capacity in response to torsional constraints, reaching peak performance when rotation is fully restricted (M=1), while resistive or driving torques reduce performance. A unique operational advantage exists in the range 0<M<1, which can be practically realized using discrete mechanical locking (M=1), and unlocking (M=0), devices. Adaptive performance in this regime is exclusive to twisting metamaterials, as conventional architected materials show no energy absorption differences between these configurations.

Experimental tests on twisting gyroid structures with 10% relative density, additively manufactured from FE7131 steel, confirmed these adaptive properties under quasi‐static and dynamic compression. Using the free‐to‐twist condition (M=0), as a reference, the twisting gyroid exhibited adaptive axial stiffness (4.1 GPa), collapse stress (19.52 MPa), and specific energy absorption (14.05 J/g), varying across the operational range −1<M<1 by −25% to +17%, −16% to +7%, and −26% to +9%, respectively. Incorporating geometric twisting slightly enhanced energy absorption, reducing peak force while maintaining equivalent specific energy absorption. Parametric finite element analyses extended these findings to 5% and 15% relative densities, accounting for AM‐induced geometric imperfections and both axial (force–displacement) and torsional (torque–rotation) responses. Adaptive energy absorption properties were mapped onto the Ashby chart as an area rather than a line and modelled with a torque‐ratio‐dependent scaling law.

Collectively, these results establish twisting gyroid structures—and, more broadly, twisting metamaterials—as a new class of adaptive crashworthy materials with tunable energy absorption, collapse stress, and stiffness controlled via torsional input. This capability represents a paradigm shift in impact protection system design, enabling active crash mitigation through actuators, snap‐action mechanisms, braking systems, or rotational inertia devices. Importantly, adaptive mechanical properties can be achieved by simply applying a twisting operation to a generic primitive lattice, providing a versatile design strategy for next‐generation energy‐absorbing metamaterials across automotive, railway, aerospace, and defence applications.

## 5 Experimental Section

### Twisting Metamaterials Design

**Figure 9:**
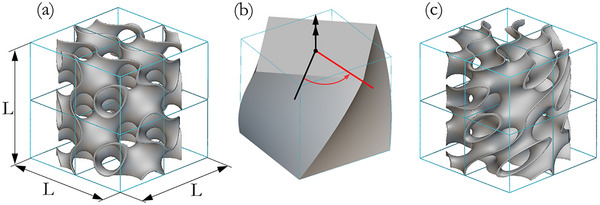
Architectural design of gyroid structures enclosed within unit‐cell of length *L* = 12 mm. a) Primitive gyroid structure, named “Gy”. b) CCW twisting operation applied to the primitive gyroid structure. c) Twisting gyroid structure, named “TwGy”.

This study designed a twisting metamaterial (right‐handed micropolar metamaterial) applying a counterclockwise (CCW) twisting operation on a primitive gyroid structure, as shown in **Figure** **9**. Preliminarily, a cylinder filled by a sheet‐based gyroid structure with an elementary cell of 6 × 6 × 6 mm was enclosed within a unit‐cell of length *L*  =  12 mm, as shown in Figure 9a. Subsequently, the primitive gyroid structure underwent a CCW twisting operation of 10 deg/mm along the cylindrical axis, Figure 9b, so that the resulting shape was a twisting sheet‐based gyroid structure, Figure 9c. The design procedure and all geometrical operations, including twisting, were carried out with nTopology software.

The unit cell and gyroid architecture dimensions were defined to ensure the best compromise between manufacturing quality and experimental testing requirements while maintaining the lowest achievable relative density. A unit cell size larger than 12×12×12 mm could not be compressed up to densification at engineering strain rate of 500 s^−1^ with the available Split Hopkinson Bar (SHB), using the first pulse. The twisting gyroid structure was designed with relative density ($\bar{\rho }$) of 10% imposing the smallest thickness achievable by the additive manufacturing technology available in the laboratory, i.e., ≈200 µm. Due to these restrictions on design parameters, the mechanical properties of twisting gyroid structure were identified by testing the unit cell. To have a fair and comprehensive comparison, the mechanical properties and energy absorption characteristics of both primitive gyroid “Gy” (primitive architected material), Figure 9a, and twisting gyroid “TwGy”, Figure 9c, structures were evaluated. Figure S5 (Supporting Information) shows a prototype of a 2 × 2 × 2 twisting gyroid structure to confirm the periodic repetition in the continuum medium of the “TwGy” unit cell.

### Additive Manufacturing

Both primitive and twisting gyroid structures, made of FE‐7131 (m4p Fe‐7131 steel powder feedstock, 20–53 µm particle size range), were manufactured by laser‐based powder bed fusion (PBF‐LB) technology using a SISMA MySint100 PM/RM, specifically developed for R&D purpose, at the AM Laboratory of the Italian National Institute of Nuclear Physics (INFN) – Gran Sasso National Laboratory (LNGS). The 3D printer was equipped with a 200 W infrared laser source and 30 µm spot laser that ensures high‐quality detail resolution for manufacturing intricate architectures, the layer thickness was 53 µm and the Volumetric Energy Density (VED) was 54.3 J/mm^3^. The PBF‐LB process parameters, summarized in the Table S6 (Supporting Information), are the results of an extensive laboratory experience aimed to provide the best geometrical accuracy, printing quality, and time efficiency, guaranteeing high structural integrity, avoiding lack of fusion, and limiting trapped unmelted powders within the gyroid “waves”.^[^ ^54^, ^55^ ^]^ The entire production process took place in an inert argon atmosphere (oxygen level < 0.1%) with a gas recirculation speed of 3 m s^−1^.

**Figure 10:**
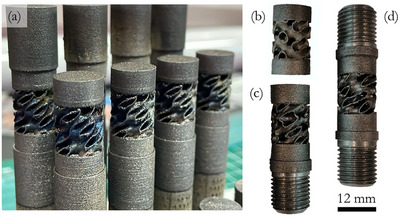
Additive manufacturing of primitive and twisting gyroid structures made of FE7131. a) As‐printed samples of gyroid structures obtained by laser‐based powder bed fusion technology. b) Sample for testing “Gy” structure. c) Sample for testing “TwGy” structure under free‐to‐twist loading configuration (“TwGy_F_”, M=0). d) Sample for testing “TwGy” structure under locking (“TwGy_L_”, M=1) and rotative (“TwGy_R_”, M=−1) loading configurations.

The gyroid samples were 3D printed with axes normal to the platform and 10 mm lifted off, using supports as shown in **Figure** **10**a, including extra dense material to be subsequently machined. “TwGy” structures were clamped to the SHB or to the universal machine through threaded ends, as shown in Figure 10d. Otherwise, when the “TwGy” structure needed to be locked to the SHB or universal machine without transmitting torque, the sample has one threaded end and one free end, as shown in Figure 10c. The “Gy” structure does not rotate during compression; thus, the associated sample has two free ends as shown in Figure 10b. Three specimens of each sample configuration were tested in quasi‐static and dynamic conditions to evaluate the experimental results’ repeatability. In addition, cylindrical samples with diameter and length equal to 4 mm were manufactured to characterize the bulk mechanical properties (FE‐7131) under quasi‐static and dynamic compression.

### Quasi‐Static Compression Test

Twisting gyroid structures were tested under combined compression and torsion using a Zwick‐Roell Z050 tensile/torsion machine equipped with a 50 kN load cell for axial loads and a 200 Nm load cell for torques (Figure S6, Supporting Information). Compression was applied at 1 mm min^−1^ under three torsional loading configurations:

- “TwGy_L_” (M=1): locking condition, in which the machine imposed zero rotations by applying CW torque.
- “TwGy_F_” (M=0): free‐to‐twist condition, with no torque transmission. The machine compressed the twisting gyroid structures while allowing free CCW rotations induced by the metamaterial architecture.
- “TwGy_R_” (M=−1): rotative condition, in which the machine rotated the twisting gyroid structures in the CCW direction at 10 deg min^−1^ during compression.

All “TwGy” samples (Figure 10d) were mounted with right‐hand threaded ends tightened to ≈50 Nm, preventing slippage during compression. This preload ensured that the applied CCW torque in the “TwGy_R_” configuration remained far below the unscrewing threshold. The primitive gyroid structures (Figure 10b) were tested under simple compression between metal plates, as in conventional architected materials. All tests were recorded using a digital camera at 2 fps. Axial displacements and rotations were extracted using a Matlab‐based image processing algorithm, which tracked axial and transverse displacements of speckled sample ends during compression.

### Dynamic Compression Test

High‐strain‐rate experiments were carried out using a dedicated Split Hopkinson Bar (SHB) setup. The pre‐stressed bar (3 m), input bar (7 m), and output bar (4.5 m) were made of titanium alloy (Ti6Al4V) with a diameter of 18 mm. Mimicking the quasi‐static configuration, samples in Figure 10d were securely screwed to the input and output bars for the “TwGy_L_” (M=1) configuration. For the “TwGy_F_” (M=0) configuration, samples in Figure 10c were screwed to the input bar and simply rested on the output bar with grease applied at the interface. Right‐hand threads prevented unscrewing and undesired torque loss during high‐speed testing. The rotative condition “TwGy_R_” (M=−1) was not tested at high strain rates. A sheet‐paper pulse shaper was used to reduce high‐frequency noise. This SHB setup enabled densification of the gyroid structures using only the first pulse, ensuring force and torque equilibrium in the sample at an engineering strain rate of ≈500 s^−1^. The novel SHB configuration allowed simultaneous measurement of axial and torsional loads, as well as compression displacements and rotations. Two axial strain gauge rosettes were placed on the input bar (midpoint) and on the output bar near the specimen, capturing the incident, reflected, and transmitted strain waves. In addition, two torsional strain gauge rosettes were installed close to the specimen ends on both input and output bars, enabling characterization of the compressive‐torsional response of twisting metamaterials at high strain rate (Figure S7, Supporting Information). Voltage signals from all strain gauges were acquired at 1 MHz using a 16‐bit DAQ card (NI PCI‐6120). Dynamic compression tests were also recorded with a high‐speed digital camera (Photron SA4, 120 000 fps). Axial displacements and rotational angles were extracted via the same Matlab‐based image processing algorithm used in quasi‐static tests. It is noteworthy that, in the “TwGy_L_” (M=1) configuration, the input shear wave was initially zero. The torsional phenomenon was generated only during compression of the twisting metamaterial; thus, torsional wavelengths had coinciding negative signs and shared the same timing as the axial waves. In contrast, in the “TwGy_F_” (M=0 configuration, the twisting gyroid structure rotated freely without torque transmission, resulting in zero torsional waves (Figure S8, Supporting Information). Forces and torques were obtained from the uniaxial wave equations described previously,^[^ ^56^ ^]^ using the axial and shear strain wave data. Representative high‐speed videos of the tests are provided (Video S1, Supporting Information).

### Micro‐Computed Tomography

X‐ray micro‐computed tomography (XµCT) allowed assessing structural integrity, printing quality, residual powder presence, and size consistency of both bulk and gyroid structures. The XµCT analysis was performed using a Zeiss Metrotom 1500 system equipped with a 0.75 mm Cu filter to enhance X‐ray beam energy transmission, reaching 11 µm voxel size with V = 199 kV and I = 130 µA. Each projection was acquired with an exposure time of 2 s and frame averaging (3 frames). A total rotation angle of 360° was used with a step size of 0.18°. The closed porosity (PC) values of the bulk and gyroid structure were quantified using Bruker SkyScan CT‐analyzer software.

### Crystallography

Information on the crystallography of bulk and gyroid‐sheet material was achieved by X‐ray diffraction (XRD), using a Bruker D8 Advance diffractometer (Bruker, Karlsruhe, Germany) with Cu‐Kα radiation, operating at V = 40 kV and I = 40 mA, in the angular range 2θ = 30–80°. Pattern analysis was performed using the DIFFRAC.EVA software package and peak indexing was carried out using the search/match facility using the PDF2 database of the International Centre for Diffraction Data (ICDD). XRD peaks shape analysis, including estimation of the lattice parameter of α‐Fe (ICDD 65–4899) bcc phase was conducted using the OriginPro 2023 software. The crystallite size was calculated using the Scherrer formula from the most intense peak α ‐Fe (110).^[^ ^57^ ^]^

### Scanning Electron Microscopy

The twisting gyroid morphology was observed with a Tescan Vega 3 scanning electron microscope (SEM), in as‐built and polished conditions. Bruker Z200 energy dispersive microanalysis (EDS) was employed to analyse the chemical composition of both bulk and gyroid‐sheet material, averaging the data taken from five different areas observed at the same magnification (1000×).

### Microhardness

Microhardness measurements assessed the homogeneity of gyroid‐sheet material mechanical properties within the gyroid structure (lines S1, S2, and S3 in Figure S3, Supporting Information). Vickers microhardness analysis was performed using a Qness 60A EVO microhardness tester, applying a load of 200 gf for a dwell time of 10 seconds.

### Numerical Modeling

The study involved a series of Finite Element (FE) analyses aimed at validating the mechanical responses of both primitive and twisting gyroid structures with $\bar{\rho }=10\%$, so that a FE parametric study could reliably explore the energy absorption capability of twisting gyroid structure across a broader range, from 5% to 15% relative densities. The numerical analyses were carried out using Abaqus/Explicit solver, importing the gyroid structures mesh from nTopology software. A modified 10‐node second‐order tetrahedral element (C3D10M) was chosen due to its higher robustness in problems involving large deformations and contact interactions.^[^ ^58^ ^]^ Initially, a mesh sensitivity analysis was carried out, and the best compromise in computational times led to an average mesh size of 0.05 mm, ensuring at least three elements along the thickness, as also demonstrated by other authors.^[^ ^46^, ^59^ ^]^ The number of elements for the gyroid structures mesh were ≈500000 elements, and an overview of the mesh model is shown in Figure S9 (Supporting Information). The contact interactions were defined with normal and tangential contact behaviors, incorporating friction properties based on a penalty factor of 0.1 and contact separation following compression loading (hard contact). To simplify the boundary conditions applications, two short beams with bulk material properties were located upper and lower gyroid structures, coupling the degree of freedom of beam node with the nodes on external gyroid surfaces, as shown in Figure S9 (Supporting Information). Therefore, the torque/rotation and force/displacement boundary conditions were directly applied to the beams. A further FE analysis was carried out on the mesh model of twisting gyroid structure obtained by XµCT‐scan to highlight the difference in numerical prediction between the effective 3D‐printed shape and CAD model.

## Conflict of Interest

The authors declare no conflict of interest.

## Supporting information



Supporting Information

Supplemental Video 1

Supplemental Video 2

Supplemental Video 3

Supplemental Video 4

Supplemental Video 5

Supplemental Video 6

## Data Availability

The data that support the findings of this study are available from the corresponding author upon reasonable request.
