# Supplementary material for: Adaptive Twisting Metamaterials
Source: Adv Mater. 2025 Oct 22;38(21):e13714. doi: 10.1002/adma.202513714 (PMC13073087; doi:10.1002/adma.202513714)
Supplement: Supplementary file 1 — Supporting Information [file ADMA-38-e13714-s001.docx]

Supporting Information

**Adaptive Twisting Metamaterials**

Mattia Utzeri*, Maria L. Gatto, Edoardo Mancini, Donato Orlandi, Daniele Cortis, Marco Sasso, and Shanmugam Kumar*

**S1. Energy density in polar-elastic material**

Splitting the total energy described in **Equation 11** in the axial internal energy ($\mathcal{L}_{a}$) and torsional internal energy ($\mathcal{L}_{t}$) as follow

$\mathcal{L=}\underset{\mathcal{L}_{a}}{\underbrace{\frac{1}{2}\sigma_{1}\varepsilon_{1}}}+\underset{\mathcal{L}_{t}}{\underbrace{\frac{1}{2}m_{1}k_{1}}}=\underset{\mathcal{L}_{a}}{\underbrace{\frac{1}{2}\left( C_{1}+\frac{{D_{1}}^{2}}{A_{1}}\mathcal{(M-}1) \right){\varepsilon_{1}}^{2}}}+\underset{\mathcal{L}_{t}}{\underbrace{\frac{1}{2}\left( \frac{\mathcal{M}{D_{1}}^{2}}{A_{1}}\mathcal{(M-}1) \right){\varepsilon_{1}}^{2}}}$ (S1)

The contributions of $\mathcal{L}_{a}$ and $\mathcal{L}_{t}$ are defined and **Figure S10** shows both trends in function of external torsional loading for a given compressive/tensile strain. Considering that the axial and torsional loadings are applied to the polar-elastic materials, both are generated by external forces (compression/tensile and CCW/CW torque, respectively) increasing the material energy density. **Figure S10** shows the $\mathcal{L}_{t}$ is negative into the range of $0< \mathcal{M<}1$, involving the torsional energy is transmitted by the materials to the environment. Therefore, the twisting metamaterial can rotate more than rotations induced by the applied torque so that the $M$ can be considered as a resistive torque in that range. Overtaking that range, ($\mathcal{M}>1 \& \mathcal{M<}0$) the external torque $M$ induces over-rotation to the material in CCW/CW directions (driving torque), increasing the torsional energy density as well as in traditional continuum mechanics (Cauchy media).

**S2. Crush Bands in Twisting Metamaterials**

The collapse mechanism associated with the crush band is illustrated in **Figure S4**, which compares numerical predictions with experimental observations. The analysis reveals that the crush band forms in the layer where the gyroid waves are unconnected (blue-shaded plane in **Figure S4d**), sandwiched between two connected layers (red-shaded plane in **Figure S4d**). The connectivity in the red-shaded layer creates a continuous network (**Figure S4e**) with higher in-plane stiffness and greater area ratio compared to the unconnected layer (**Figure S4f**). This difference in structural stiffness localizes the collapse within the weaker, unconnected layer. As a result, the layer-by-layer variation in area ratio and in-plane stiffness directly influences crush band formation, triggering localized deformation and collapse during compression (see **Video S3** and **S5**). Following the initiation of the crush band, the ductility of the gyroid-sheet material plays a key role in the post-collapse phase, enabling the complete folding of the gyroid waves. **Figure S4b** highlights extensive plastic deformation within the crush bands, confirming plastic yielding as a governing collapse mechanism. During post-collapse deformation, the structure undergoes a sequential collapse, with crush bands localizing progressively around unconnected regions until full densification is reached.

**Supplementary Figures**

Figure S1: Experimental and analytical prediction with Johnson-Cook model of mechanical response of bulk material FE-7131 under quasi-static and dynamic compression.


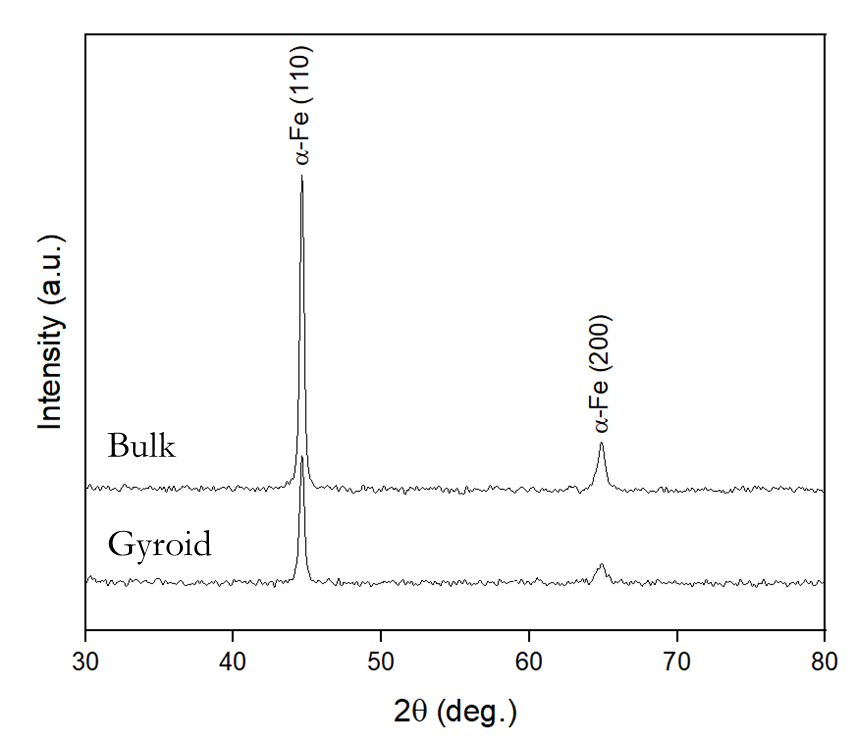


Figure S2: XRD patterns of bulk and gyroid-sheet materials (FE-7131)


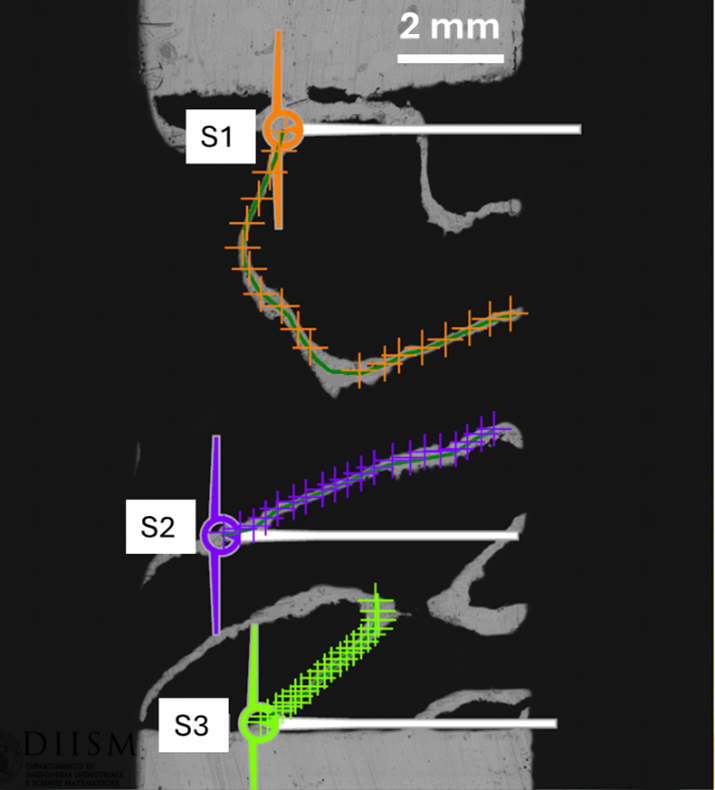


Figure S3: Schematics of microhardness measurements across twisting gyroid structure.


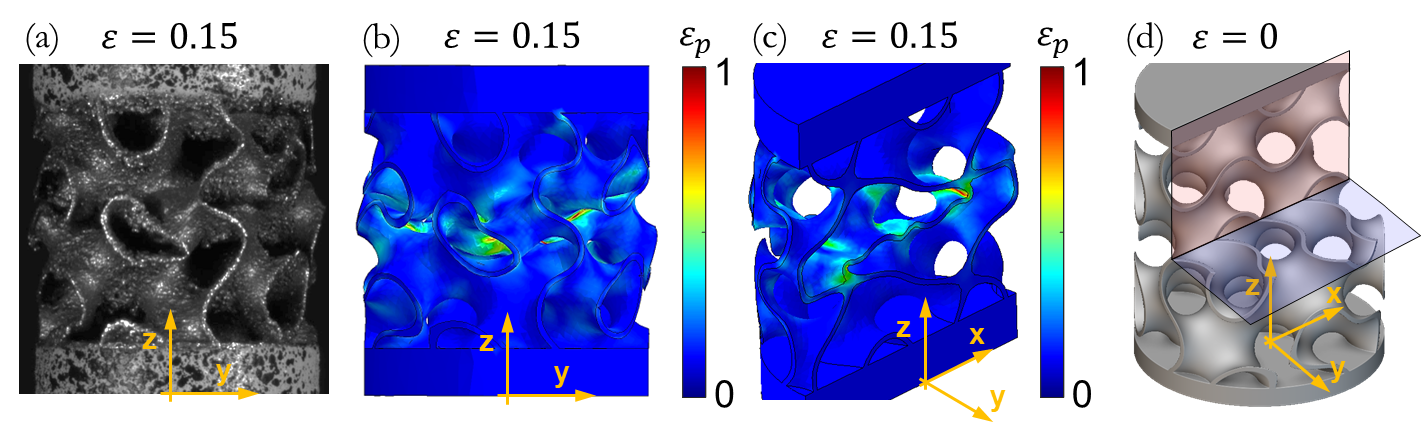


Figure S4: Crush band initiation in gyroid structures with $\bar{\rho}$= 10%. (a) Experimental vs FE predictions (b) of crush band shape and local equivalent plastic strain ($\varepsilon_{p}$). (c) Gyroid structure interior visualizing the collapsed gyroid waves and local $\varepsilon_{p}$. (d) Interior of undeformed gyroid structure visualizing the location where the crush band occurred (blue shaded plane). (e) Connected gyroid waves layer. (f) Unconnected gyroid waves layer.


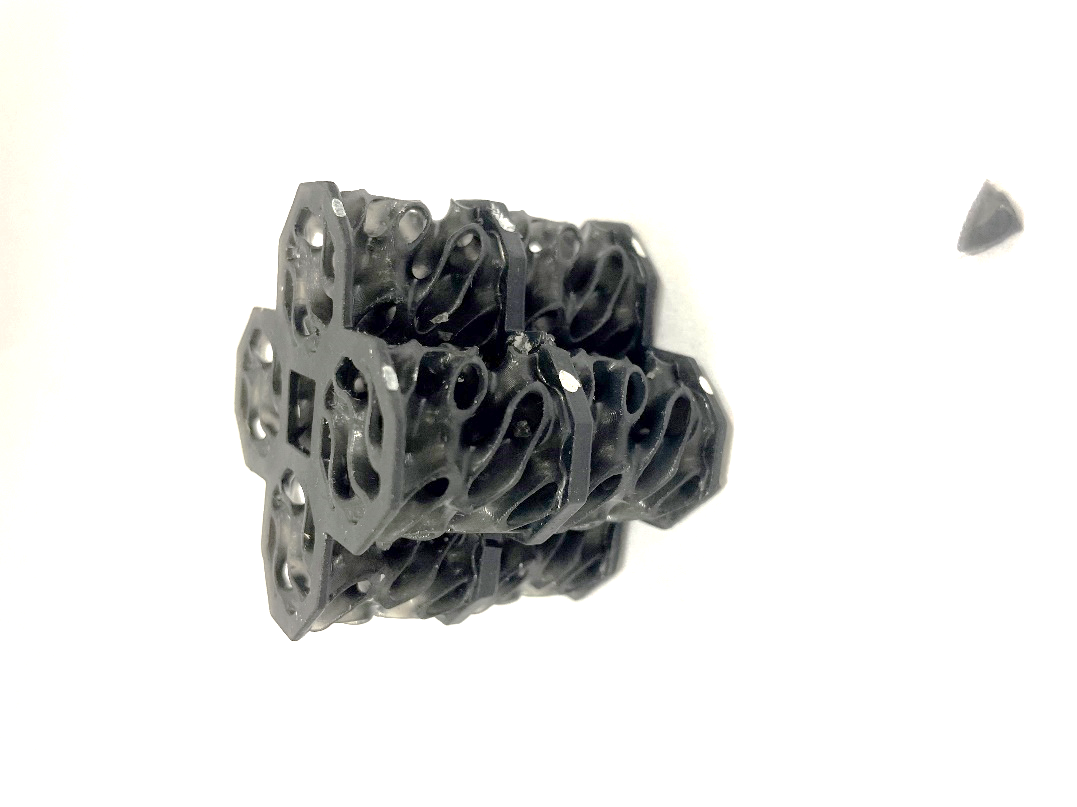


Figure S5: Additively manufactured prototype of 2x2x2 twisting gyroid structured made of polyurethane.


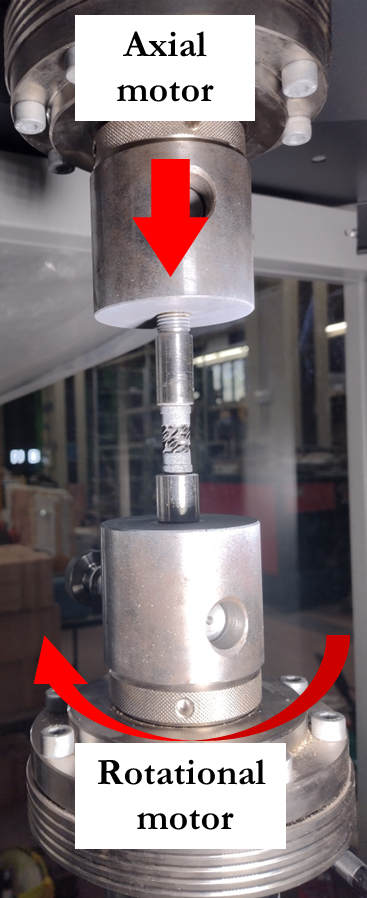


Figure S6: Experimental set up for characterize the quasi-static compressive response of twisting gyroid structures. “TwGy_R_” configuration ($\mathcal{M=-}1$), sample was screwed to the universal machine and simultaneously compressed and rotated.


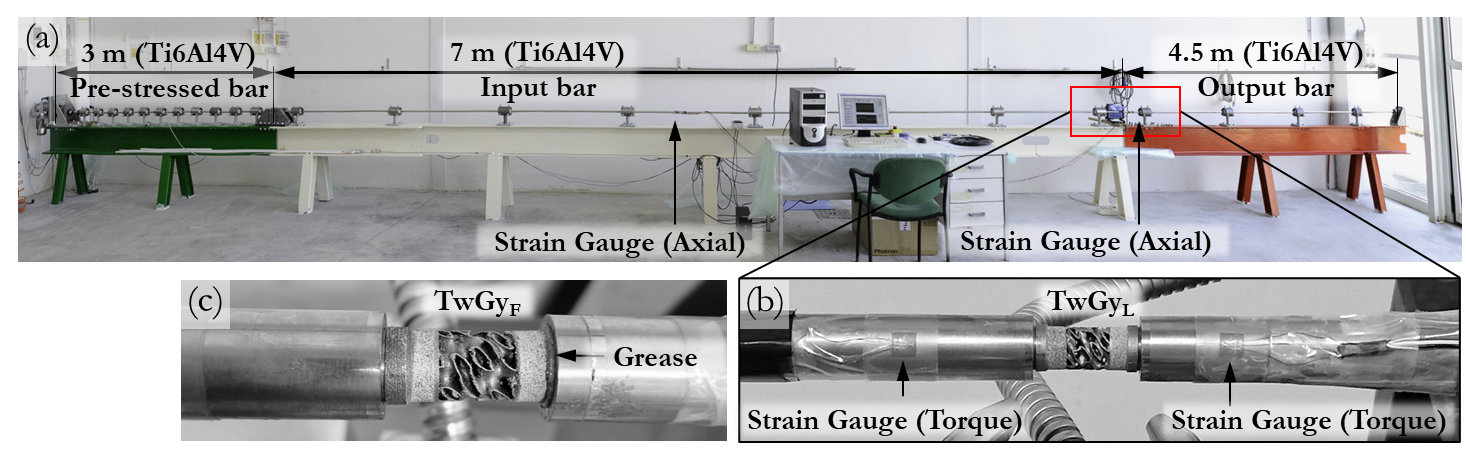


Figure S7: Experimental set up for characterize the compressive response of twisting metamaterials at high strain rate. (a) Modified split Hopkinson bar composed by pre-stressed, input, and output titanium bars. (b) “TwGy_L_” configuration ($\mathcal{M=}1$), sample was screwed to the input and output bar. Additional strain gauges for torque measurement were installed close to the sample. (c) “TwGy_F_” configuration ($\mathcal{M=}0$), sample was screwed to the input bar and simply leaned on the output bars, placing grease.


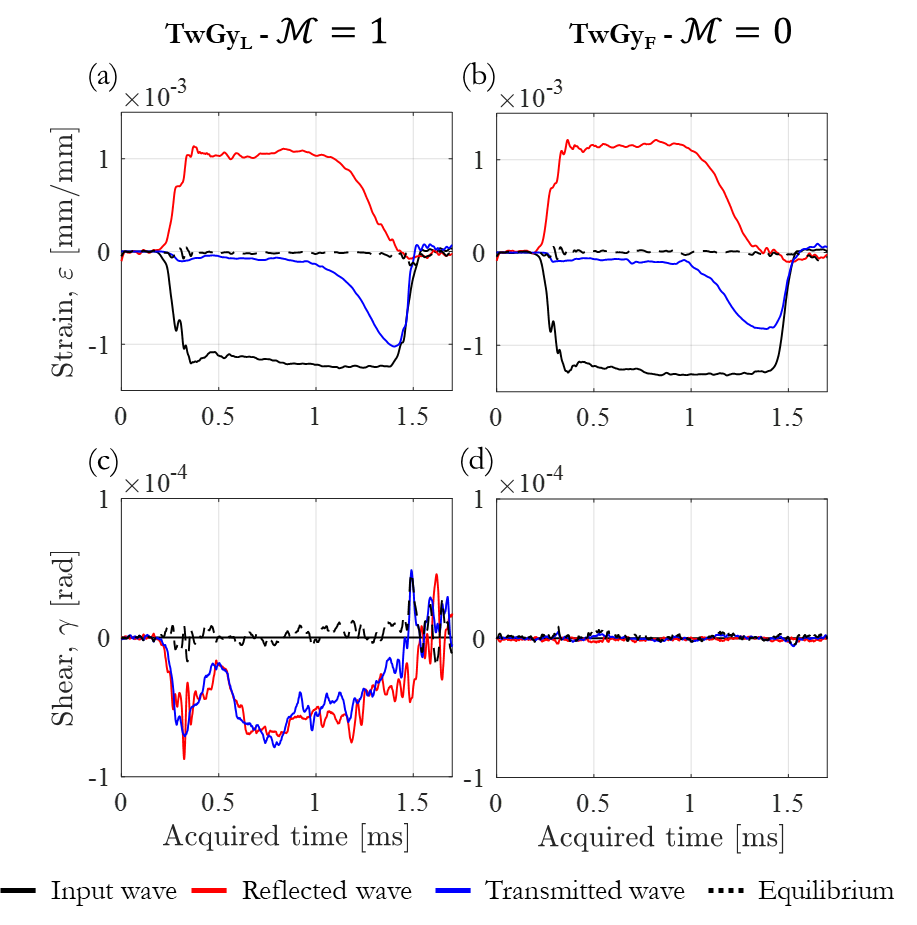


Figure S8: Axial and torsional strain gauges signals acquired during high-speed impact test on twisting metamaterials with proposed SHB set up. Input, transmitted and reflected axial strain waves in “TwGy_L_” (a) and “TwGy_F_” (b) configurations. Input, transmitted and reflected shear waves in “TwGy_L_” (c) and “TwGy_F_” (d) configurations.

**
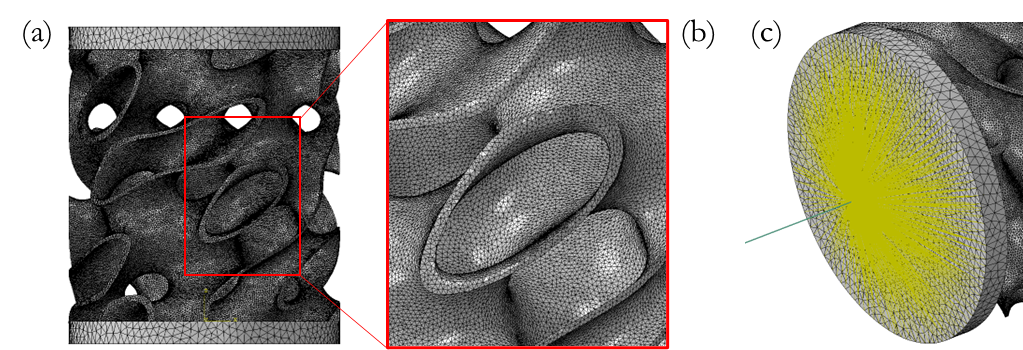
**

Figure S9: Overview of mesh model of twisting gyroid structure (a) for FE simulation. (b) Detail of mesh into gyroid wave. (c) Nodes connection between planar surface of mesh model and the short beam located at the upper side.


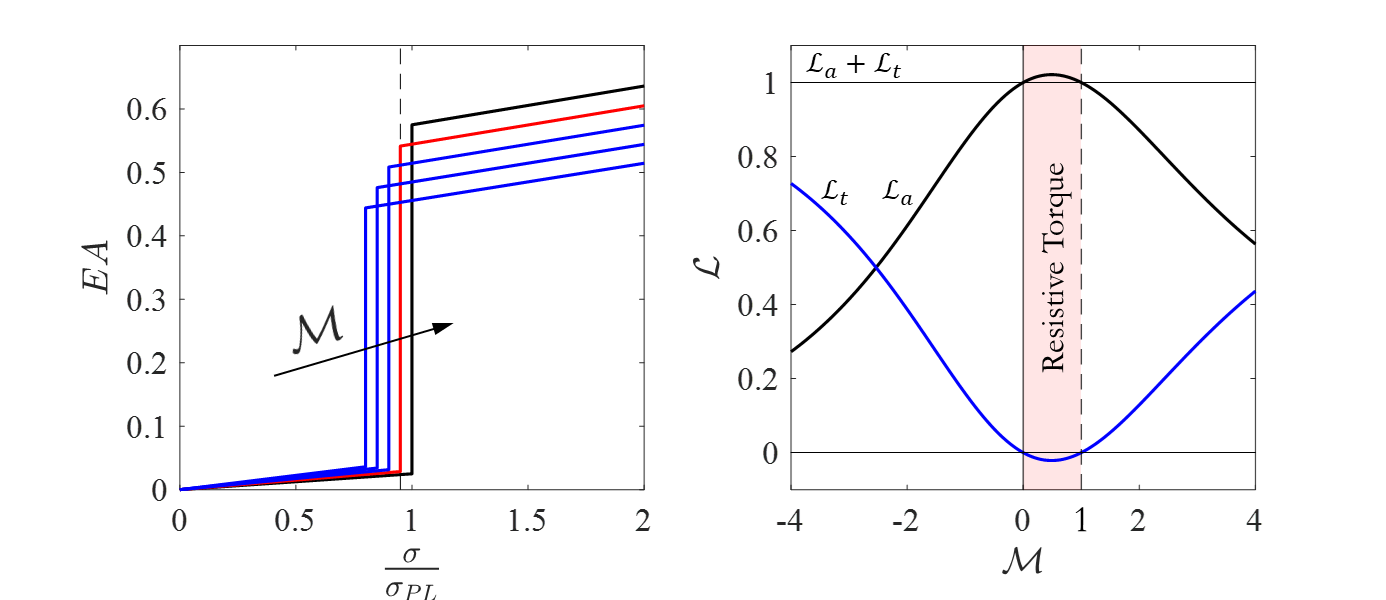


Figure S10: Influence of torque ratio on energy density components in polar-elastic material at given strain.


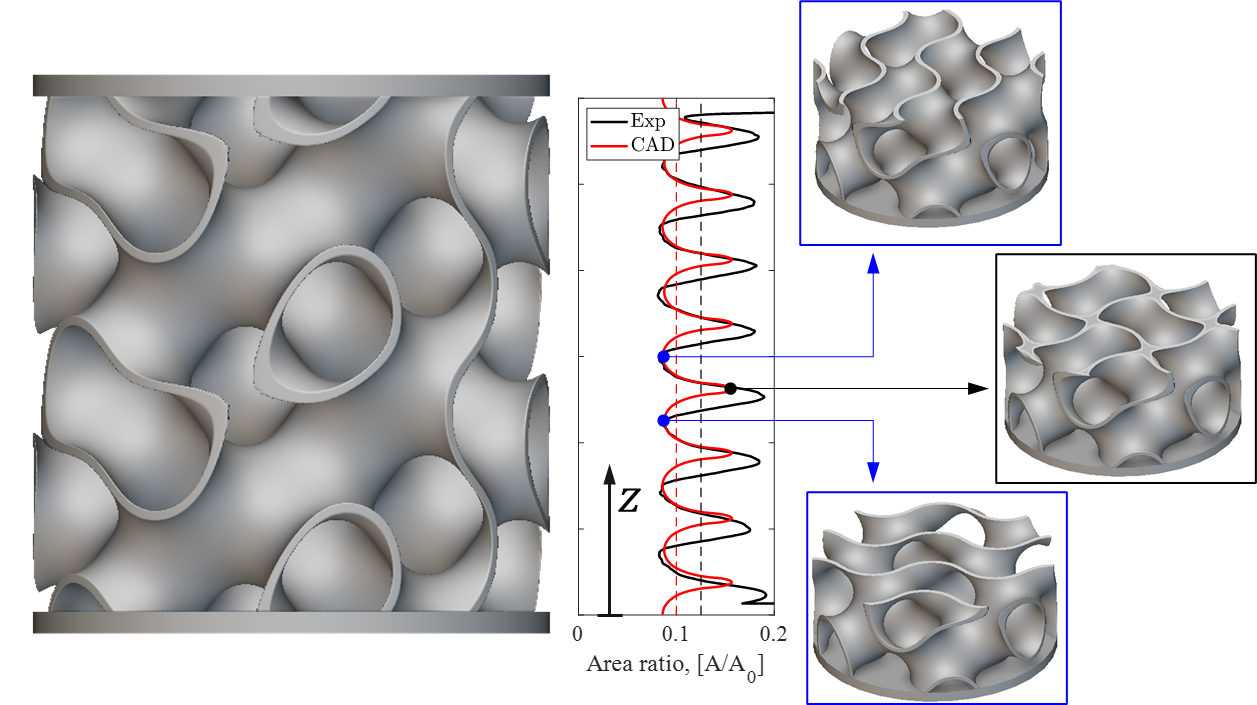


Figure S11: Overview area ratio fluctuations in gyroid structure along the vertical direction because of layer with connected gyroid waves (high values) and unconncected gyroid waves (low values).

**Supplementary Tables**

Table S1: Loading configurations for calibrating the constitutive parameters of polar-elastic material in Cosserat continuum mechanics.

| Configuration | Torque ratio | Axial load | Torsional load |
| --- | --- | --- | --- |
| TwGy_L_ | $\mathcal{M}=1$ | $\sigma_{1}=C_{1}\varepsilon_{1}$ | $m_{1}=D_{1}\varepsilon_{1}$ |
| TwGy_F_ | $\mathcal{M}=0$ | $\sigma_{1}=\left( C_{1}-\frac{{D_{1}}^{2}}{A_{1}} \right)\varepsilon_{1}$ | $A_{1}k_{1}=-D_{1}\varepsilon_{1}$ |

Table S2: Johnson-Cook parameters for bulk FE-7131 material.

| $A_{JC}$  [MPa] | $B_{JC}$  [MPa] | $n_{JC}$  [-] | $C_{JC}$  [-] | $\dot{\varepsilon_{0}}$  [1/s] | $m_{JC}$  [-] |
| --- | --- | --- | --- | --- | --- |
| 680 | 415 | 0.22 | 0.055 | 300 | 1.05 |

Table S3: Chemical composition of bulk and gyroid-sheet material (FE-7131).

|  | 𝐶 | 𝐶r | Si | 𝑀n | Fe |
| --- | --- | --- | --- | --- | --- |
| Bulk material | 0.14^*^ | 1.07 | 0.35 | 0.72 | Bal. |
| Gyroid-sheet material | 0.14^*^ | 1.14 | 0.45 | 0.70 | Bal. |
| *From technical datasheet | | | | | |

Table S4: Results of peak shape analysis conducted on α-Fe (110) peak for bulk and gyroid structure. 2θ (◦) – peak angular position; FWHM (◦) - Full width at half maximum; L – average size of crystallites from Scherrer equation; a_exp_ – experimental value of lattice parameter; Δa/a_nom_ – relative difference (unit: %) between experimental and nominal lattice parameter (Δa = a_exp_ – a_nom_).

| Sample | Peak | 2θ  [°] | FWHM  [°] | L  [nm] | a_exp_  [nm] | a/a_nom_  [%] |
| --- | --- | --- | --- | --- | --- | --- |
| Bulk | α-Fe (110) | 44.6234 ± .0005 | 0.363 ± 0.001 | 23.41 ± 0.08 | 3.5142 ± 0.0003 | 0.23 |
| Gyroid-sheet | α-Fe (110) | 44.621 ± 0.001 | 0.399 ± 0.002 | 21.26 ± 0.12 | 3.5144 ± 0.0006 | 0.23 |

Table S5: Theoretical, experimental and numerical values of quasi-static energy absorption performances of twisting gyroid structures.

| Sample  code | Unit cell  topology | $\bar{\rho}$  [-] | $\mathcal{M}$  [-] | W  [MJ/m^3^] | W*  [MJ/m^3^] |
| --- | --- | --- | --- | --- | --- |
| TwGy_L_ | Twist Gy. | 0.1 | 1 | 12.05±0.26 | 12.11 |
| TwGy_F_ | Twist Gy. | 0.1 | 0 | 11.09±0.20 | 11.16 |
| TwGy_R_ | Twist Gy. | 0.1 | -1 | 8.12±0.17 | 8.30 |
| TwGy_L_ | Twist Gy. | 0.05 | 1 | 3.14 | 2.82 |
| TwGy_F_ | Twist Gy. | 0.05 | 0 | 2.81 | 2.60 |
| TwGy_R_ | Twist Gy. | 0.05 | -1 | 1.52 | 1.93 |
| TwGy_L_ | Twist Gy. | 0.15 | 1 | 27.79 | 28.38 |
| TwGy_F_ | Twist Gy. | 0.15 | 0 | 24.96 | 26.14 |
| TwGy_R_ | Twist Gy. | 0.15 | -1 | 18.21 | 19.44 |
| *Equation (15) with $C=1525$MJ/m^3^, $\alpha=2.10$, and $\beta=-121$ MJ/m^3^. | | | | | |

Table S6: PBF-LB process parameter for FE-7131: laser power ($P_{AM}$), laser scanning speed ($S_{AM}$), hatch distance ($h_{AM}$) layer thickness ($L_{AM}$), and Volumetric Energy Density (VED) are.

| $P_{AM}$  [W] | $S_{AM}$  [mm/s] | $h_{AM}$  [μm] | $L_{AM}$  [μm] | VED [J/mm^3^] |
| --- | --- | --- | --- | --- |
| 175 | 1150 | 70 | 40 | 54.3 |
